# Analysis of the Total Biflavonoids Extract from *Selaginella doederleinii* by HPLC-QTOF-MS and Its In Vitro and In Vivo Anticancer Effects

**DOI:** 10.3390/molecules22020325

**Published:** 2017-02-20

**Authors:** Hong Yao, Bing Chen, Yanyan Zhang, Huigen Ou, Yuxiang Li, Shaoguang Li, Peiying Shi, Xinhua Lin

**Affiliations:** 1 Department of Pharmaceutical Analysis, School of Pharmacy, Fujian Medical University, Fuzhou 350122, China; yauhung@126.com (H.Y.); chanbing@foxmail.com (B.C.); zhangyyfjmu@sina.com (Y.Z.); hgou2014@sina.com (H.O.); yuxiangli2014@sina.com (Y.L.); lsg941@sina.com (S.L.); 2 Department of Traditional Chinese Medicine Resource and Bee Products, Fujian Agriculture and Forestry University, Fuzhou 350002, China

**Keywords:** *Selaginella doederleinii*, HPLC-ESI-QTOF MS/MS, biflavonoids, murine B16 melanoma cell, mouse lewis lung cancer (LLC) cell, LLC mouse model

## Abstract

*Selaginella doederleinii* Hieron has been traditionally used as a folk antitumor herbal medicine in China. In this paper, the phytochemical components of the total biflavonoids extract from *S. doederleinii* were studied by using high-performance liquid chromatography coupled with electrospray ionization quadrupole time-of-flight mass spectrometry (HPLC-ESI-QTOF MS/MS) in negative ion mode, and their in vitro and in vivo anticancer effects were evaluated. Four types of biflavonoids from *S. doederleinii*, including IC3′–IIC8′′, IC3′–IIC6′′, IC3′–IIC3′′′, and C–O linked biflavonoids were examined originally using QTOF MS/MS. The fragmentation behavior of IC3′–IIC3′′′ linked biflavonoids was reported for the first time. A total of twenty biflavonoids were identified or tentatively characterized and eight biflavonoids were found from *S. doederleinii* for the first time. Furthermore, the 3-(4,5-Dimethyl-2-thizolyl)-2,5-diphenyltertazolium bromide (MTT) assay and xenograft model of mouse lewis lung cancer(LLC) in male C57BL/6 mice revealed favorable anticancer properties of the total biflavonoids extracts from *S. doederleinii*. The results of this work could provide useful knowledge for the identification of biflavonoids in herbal samples and further insights into the chemopreventive function of this plant.

## 1. Introduction

*Selaginella doederleinii* Hieron, belonging to the *Selaginella* genus of the *Selaginellaceae* family, has been traditionally used as a folk medicine/antitumor agent in China for the treatment of various cancers. *S. doederleinii* and pork meat are often cooked together and eaten for the treatment of pneumonia, acute tonsillitis, and ocular conjunctivitis in China [1]. A few components have been isolated from this plant genus, including flavonoids, lignans, polysaccharides, and so on [2], some of which have been reported to possess anti-diabetic, anti-inflammation, anti-oxidative, and anti-cancer activities [3,4,5,6,7,8,9,10,11]. Modern pharmacological studies showed that the ethanol extract of *S. doederleinii* could induce human nasopharyngeal carcinoma cell (CNE) apoptosis [5] and because of abundant biflavonoids, the ethyl acetate extract had more powerful anti-cancer activities than the ethanol extract for *S. doederleinii* [6,7,8,9,10,11]. Our recent studies demonstrated that the biflavonoids in *S. doederleinii* were concentrated mostly in the ethyl acetate extract, and the total biflavonoids could reduce the expression of antigen Ki67, decrease tumor microvessel density (MVD), and significantly inhibit the tumor growth, and did not show apparent oral acutetoxicity in healthy mice [9]. All these indicate that the total biflavonoids in *S. doederleinii* possess favorable functional propertiesin human health. However, the component information on the total biflavonoids of *S. doederleinii* is still insufficient and the functional usage in human health needs supporting evidence as much as possible.

High-resolution tandem mass spectrometry can provide elemental composition information of both the precursor and product ions, which is very helpful in fragmentation pattern deduction [12,13,14,15,16,17] and structural characterization for natural products, such as polyphenols [18,19], flavonoids [19,20,21], alkaloids [22], and terpenes [18,21]. Using this technique, the fragmentation behaviors in the positive ion mode of biflavonoids in the *Selaginella* genus have been studied [23,24], which will aid in the elucidation of unknown compounds of this kind. However, until now, the fragmentation patterns in negative electrospray ionization ((−)ESI) of biflavonoids in the *Selaginella* genus have been rarely reported. Since it appeared more selective and more sensitive for liquid chromatography-mass spectrometry analysis of flavonoids in (−)ESI mode [25], the fragmentation rules under (−)ESI conditions of biflavonoids in the *Selaginella* genus require further consideration.

This paper aims to identify the biflavonoid components in *S. doederleinii* using high performance liquid chromatography of quadrupole time of flight-tandem mass spectrometry (HPLC-QTOF MS/MS) in (−)ESI mode. Furthermore, the in vitro and in vivo anti-cancer effects of the total biflavonoids (ethyl acetate extract) of *S. doederleinii* were evaluated using the MTT assay and a xenograft model of mouse lung cancer LLC in male C57BL/6Slac mice. The presented work could serve as a useful reference for the identification of biflavonoids in herbal samples and provide further insights into the chemopreventive function of this plant.

## 2. Results and Discussion

### 2.1. (−)ESI-QTOF MS/MS Analysis of Biflavonoid Reference Compounds

Eight biflavonoid standards obtained from *S. doederleinii* could be generally classified into four categories, viz. IC3′–IIC8′′ (amentoflavone, heveaflavone, and 7,4′,7′′,4′′′-tetra-*O*-methyl-amentoflavone), IC3′–IIC6′′ (robustflavone), IC3′–IIC3′′′ (2′′,3′′-dihydrogen-3′,3′′′-biapigenin and 3′,3′′′-binaringenin), and C–O (delicaflavone and chrysocauloflavone I) linked biflavonoids. In order to investigate the fragmentation behaviors of biflavonoids under (−)ESI conditions, the mass accuracy measurements of precursor ions and product ions were developed with a QTOF MS instrument. All biflavonoid references were ionized and showed [M − H]^−^ ions in (−)ESI mode. These precursor ions dissociated at low collision-induced dissociation (CID) energy and gave the fragments in the MS/MS spectra, which are summarized in Table S1 in the Supplementary Materials.

#### 2.1.1. Characterization of IC3′–IIC8′′ Linked Biflavones

**Figure 1:**
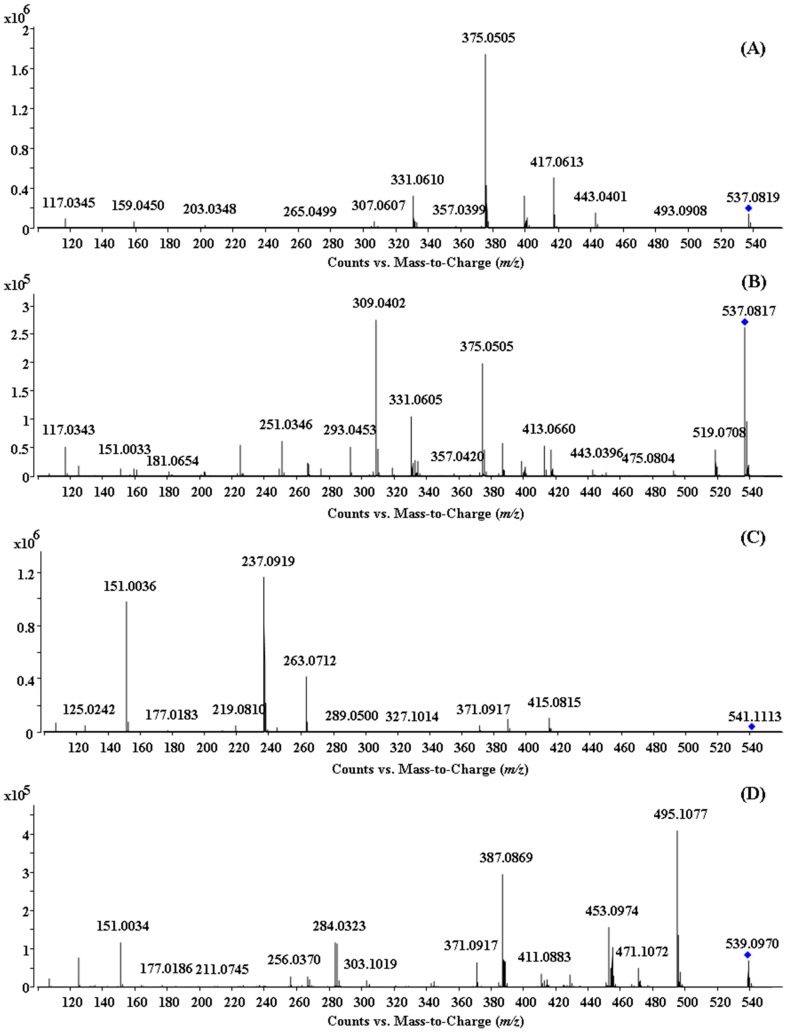
(−)ESI-QTOF MS/MS spectra of four biflavonoid reference compounds: (**A**) amentoflavone; (**B**) robustflavone; (**C**) 3′,3′′′-binaringenin; (**D**) chrysocauloflavone I.

The [M − H]^−^ ion of amentoflavone at *m*/*z* 537 produced the [M − H − C_6_H_6_O]^−^ ion at *m*/*z* 443, [M − H − C_7_H_4_O_2_]^−^ ion at *m*/*z* 417, [M − H − C_7_H_6_O_3_]^−^ ion at *m*/*z* 399, [M – H − C_9_H_6_O_3_]^−^ ion at *m*/*z* 375, [M − H − C_10_H_6_O_5_]^−^ ion at *m*/*z* 331, and C_8_H_5_O^−^ ion at *m*/*z* 117 in the MS^2^ spectrum. The *m*/*z* 375 ion corresponded to the base peak, which indicated that the product ion underwent a retrocyclization fragmentation involving the 0 and 4 bonds on flavonoid part II, while the *m*/*z* 331 ion corresponded to the ^0,4^IIA^−^ − CO_2_ ion. The *m*/*z* 417 ion was attributed to the ^0,2^IIA^−^ ion, while the *m*/*z* 399 ion corresponded to the ^0,2^IIA^−^ − H_2_O ion. In addition, the *m*/*z* 443 ion resulted from the neutral loss of phenol on flavonoid part II, and the *m*/*z* 117 ion was attributed to the ^1,3^IIB^−^ ion through the retro Diels-Alder (RDA) reaction. The primary product ion mass spectrum of amentoflavone is shown in Figure 1A, and the proposed fragmentation pathway is shown in Figure S1 in the Supplementary Materials. The nomenclature adopted for the fragments was also illustrated in the structure [23].

The [M − H]^−^ ion at *m*/*z* 579 of heveaflavone (7,7′′,4′′′-tri-*O*-methyl-amentoflavone) yielded the [M − H − C_10_H_8_O_3_]^−^ ion at *m*/*z* 403 corresponding to the ^0,4^IIA^−^ ion and ^0,4^IIA^−^ − CH_3_• ion at *m*/*z* 388 in the MS^2^ spectrum.

Similarly, the [M − H]^−^ ion at *m*/*z* 593 of 7,4′,7′′,4′′′-tetra-*O*-methyl-amentoflavone generated the [M − H − C_2_H_5_OH]^−^ ion at *m*/*z* 547, and the [M − H − 2CH_3_•]^−^ ion at *m*/*z* 563 corresponded to the base peak in the MS^2^ spectrum.

Generally, the most useful fragmentation in (−)ESI mode in terms of amentoflavone-type biflavones is that involving the cleavage of the C–ring of flavonoid part II at position 0/4, which has also been viewed as a diagnostic fragmentation for this type of biflavone in the positive ion mode [24]. The cleavage of the C–ring of flavonoid part II at position 0/2 and 1/3 could also be observed. When the hydroxy groups were substituted with methoxyl groups on this type of biflavone structure, the consecutive loss of methyl groups occurred. The neutral loss of H_2_O or C_2_H_5_OH often happened at the 4′-hydroxy and/or methoxyl group together with the space-adjacent 7′′-hydroxy and/or methoxyl group, similar with the fragmentation patterns in positive ion mode [24].

#### 2.1.2. Characterization of IC3′–IIC6′′ Linked Biflavones

**Figure 2:**
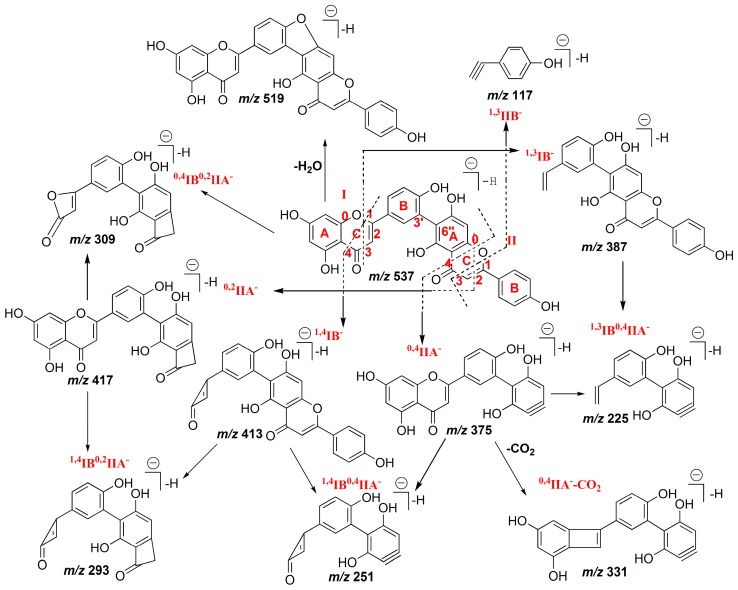
Proposed fragmentation pathway of robustflavone in (−)ESI mode.

The ESI-MS^2^ fragmentation pathways of IC3′–IIC6′′ linked biflavones, such as robustflavone, had similarities and differences compared with amentoflavone-type biflavones. In the MS^2^ spectrum of the [M − H]^−^ ion of robustflavone, the [M − H − H_2_O]^−^ ion at *m*/*z* 519, ^0,4^IIA^−^ ion at *m*/*z* 375, ^0,4^IIA^−^ − CO_2_ ion at *m*/*z* 331, ^0,2^IIA^−^ ion at *m*/*z* 417, and the ^1,3^IIB^−^ ion at *m*/*z* 117 were observed. The C-ring fragmentation also occurred on flavonoid part I, including the ^1,4^IB^−^ ion at *m*/*z* 413, ^1,3^IB^−^ ion at *m*/*z* 387, ^0,4^IB^0,2^IIA^−^ ion at *m*/*z* 309, ^1,4^IB^0,2^IIA^−^ ion at *m*/*z* 293, ^1,4^IB^0,4^IIA^−^ ion at *m*/*z* 251, and the ^1,3^IB^0,4^IIA^−^ ion at *m*/*z* 225. The product ion mass spectrum of robustflavone is shown in Figure 1B, and the proposed fragmentation pathway is shown in Figure 2.

Compared with the IC3′–IIC8′′ linked biflavones, the chances are greater for the cleavage of the C–ring to occur on flavonoid part I for the IC3′–IIC6′′ linked biflavones, such as at position 1/4 and 1/3. Among them, the retrocyclization fragmentation involving the 1 and 4 bonds on flavonoid part I and leading to the ^1,4^IB^−^ ion in the MS^2^ spectrum, could be used to distinguish robustaflavone-type biflavones from amentoflavone-type biflavones, which was consistent with the diagnostic fragmentation of robustaflavone in positive ion mode [24].

#### 2.1.3. Characterization of IC3′–IIC3′′′ Linked Biflavonoids

The [M − H]^−^ ion of 2′′,3′′-dihydrogen-3′,3′′′-biapigenin produced the ^1,3^IIB^−^ ion at *m*/*z* 387 undergoing RDA rupture corresponding to the base peak, ^0,4^IIB^−^ − H_2_O ion at *m*/*z* 413, and the ^1,3^IIB^−^ − H_2_O ion at *m*/*z* 369 in the MS^2^ spectrum. The proposed fragmentation pathway of 2′′,3′′-dihydrogen-3′,3′′′-biapigenin is shown in Figure S2 in the Supplementary Materials.

**Figure 3:**
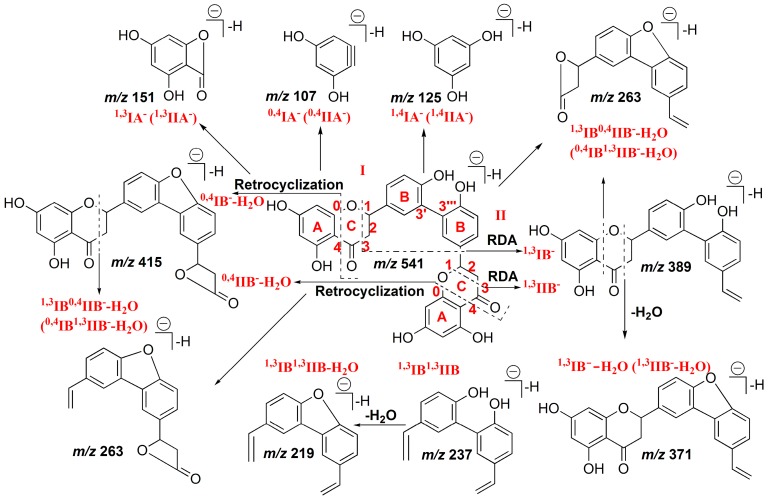
Proposed fragmentation pathway of 3′,3′′′-binaringenin in (−)ESI mode.

Interestingly, the structure of 3′,3′′′-binaringenin has two identical flavonoids linked via IC3′–IIC3′′′, thus the fragmentations involving cleavage of the C−ring could have occurred in part I, as well as in part II. The major pathways observed are the RDA reaction at position 1/3, retrocyclization fragmentation involving the 0 and 4 bonds, and the neutral loss of H_2_O, similar with that of 2′′,3′′-dihydrogen-3′,3′′′-biapigenin. The product ion mass spectrum of 3′,3′′′-binaringenin is shown in Figure 1C, and the proposed fragmentation pathway is shown in Figure 3.

Hence, the fragmentation routes involving the cleavage of the C–ring at positions 1/3 and 0/4 and leading to the ^1,3^IB^−^ (^1,3^IIB^−^) and ^0,4^IB^−^ (^0,4^IIB^−^) ions are the main pathways of IC3′–IIC3′′′ linked biflavonoids. Furthermore, the cleavage at positions 1/3 and 0/4 occurred more easily in the flavanone part than in flavone part, producing characteristic product ions with small molecular weights, such as the ^1,3^IB^0,4^IIB^−^ − H_2_O (^0,4^IB^1,3^IIB^−^ − H_2_O) ion at *m*/*z* 263, ^1,3^IB^1,3^IIB^−^ ion at *m*/*z* 237, and ^1,3^IB^1,3^IIB^−^ − H_2_O ion at *m*/*z* 219.

#### 2.1.4. Characterization of C–O Linked Biflavonoids

**Figure 4:**
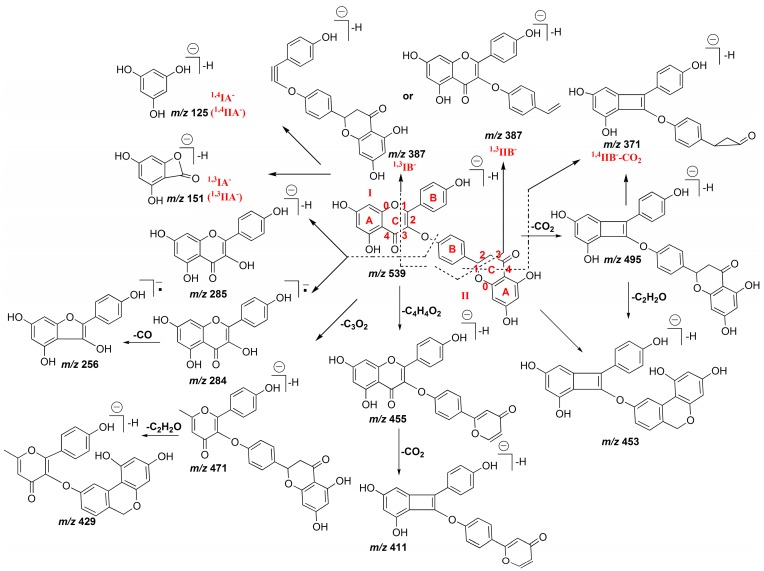
Proposed fragmentation pathway of chrysocauloflavone I in (−)ESI mode.

The fragmentations of chrysocauloflavone I and delicaflavone demonstrated a large distinction with the former types of biflavonoids discussed above because their two flavonoid parts are connected via a C–O bond and not a C–C bond. For chrysocauloflavone I, the *m*/*z* 285, 284, and 256 product ions resulted from the rupture of the connective C–O bond. In addition, the [M − H]^−^ ion of chrysocauloflavone I also generated the [M − H − CO_2_]^−^ ion at *m*/*z* 495 corresponding to the base peak, [M − H − C_3_O_2_]^−^ ion at *m*/*z* 471, [M − H − C_4_H_4_O_2_]^−^ ion at *m*/*z* 455, [M − H − CO_2_ − C_2_H_2_O]^−^ ion at *m*/*z* 453, [M − H − C_3_O_2_ − C_2_H_2_O]^−^ ion at *m*/*z* 429, [M − H − C_4_H_4_O_2_ − CO_2_]^−^ ion at *m*/*z* 411, ^1,3^IB^−^ or ^1,3^IIB^−^ ion at *m*/*z* 387, ^1,4^IIB^−^ − CO_2_ ion at *m*/*z* 371, ^1,3^IA^−^ or ^1,3^IIA^−^ ion at *m*/*z* 151, and the ^1,4^IA^−^ or ^1,4^IIA^−^ ion at *m*/*z* 125. Among them, the neutral loss of C_2_H_2_O occurs on the C–ring followed by a new cyclization involving the B–ring, and the C_3_O_2_ loss implies the β–dihydroxy configuration displayed by the A–ring [25]. Moreover, the fragmentation patterns of neutral losses of CO, C_2_H_2_O, CO_2_, and C_3_O_2_ obtained from the corresponding [M − H]^−^ ion of chrysocauloflavone I, the RDA reaction at position 1/3, and the retrocyclization fragmentation involving the 0 and 4 bonds are identical to those of isosakurametin, a 4′-methoxylated flavanone, which support the proposed fragments [25]. The product ion mass spectrum of chrysocauloflavone I is shown in Figure 1D, and the proposed fragmentation pathway is shown in Figure 4.

Similarly, for delicaflavone, the *m*/*z* 284, 269, 256, and 255 product ions were ascribed to the rupture of the connective C–O bond. The *m*/*z* 413 ion corresponded to the ^1,4^IIB^−^ ion, the *m*/*z* 319 ion was attributed to the ^1,3^IB^−^ − C_3_O_2_ (^1,3^IIB^−^ − C_3_O_2_) ion, the *m*/*z* 227 ion corresponded to the ^1,3^IB^−^ − C_3_O_2_ − C_2_H_2_O (^1,3^IIB^−^ − C_3_O_2_ − C_2_H_2_O) ion, while the *m*/*z* 151 and 107 ions were attributed to the ^1,3^IA^−^ (^1,3^IIA^−^) and ^0,4^IIA^−^ (^0,4^IIA^−^) ions, respectively. And the other ions including *m*/*z* 227, 193, 192, 175, and 147 ions were mostly related to the cleavage of flavonoid skeletons. The proposed fragmentation pathway of delicaflavone was shown in Figure S3 in the Supplementary Materials.

Therefore, for C–O linked biflavonoids, the most specific and predominant diagnostic ions are a series of product ions at *m*/*z* 284, 269, 256, and 255 for delicaflavone, and at *m*/*z* 285, 284, 256, for chrysocauloflavone I, which result from the rupture of the connective C–O bond, similar with the fragmentation pattern in the positive ion mode [24]. Meanwhile, the C_2_H_2_O and C_3_O_2_ losses are more likely to occur for C–O linked biflavonoids.

### 2.2. HPLC-ESI-QTOF MS/MS Analysis of the Biflavonoids from S. doederleinii

**Figure 5:**
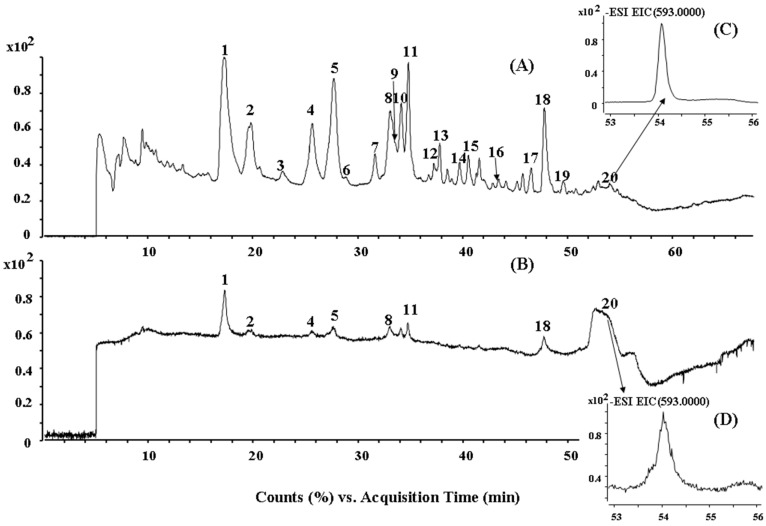
TIC chromatograms in the negative ion mode of (**A**) an extract of *S. doederleinii* and (**B**) the eight reference compounds, the extracted ion chromatograms (EIC, *m*/*z* 593) in the negative ion mode of (**C**) an extract of *S. doederleinii* and (**D**) the 8 reference compounds.

Total ion current (TIC) chromatograms in (–)ESI mode of the ethyl acetate extract of *S. doederleinii* and the eight reference compounds are shown in Figure 5. The data of t_R_, [M − H]^−^ ions and molecular weight (MW) are summarized in Table 1. MS/MS spectra of the constituents detected in *S. doederleinii* extracts are also summarized. Of them, twelve MS/MS spectra data are listed in Table 2 and the other eight components MS/MS spectra data are accordant with those of their references and listed in Table S1 in the Supplementary Materials. According to the MS data and the literature [23,26,27,28,29], twenty biflavonoids were identified or tentatively characterized, including eight IC3′–IIC8′′, one I2′–IIC8′′, two IC3′–IIC6′′, four IC3′–II3′′′,and five C–O linked biflavonoids. Peaks 1, 2, 4, 5, 8, 11, 18, and 20 were confirmed to be amentoflavone, robustaflavone, 2′′,3′′-dihydro-3′,3′′′-biapigenin, 3′,3′′′-binaringenin, delicaflavone, chrysocauloflavone I, heveaflavone, and 7,4′,7′′,4′′′-tetra-*O*-methyl-amentoflavone, respectively, by comparison of their t_R_, MS, and MS/MS data with those of the eight reference compounds.

#### 2.2.1. Characterization of IC3′–IIC8′′ Linked Biflavonoids from *S. doederleinii*

Peak 6 at a retention time (t_R_) of 28.9 min gave an [M − H]^−^ ion at *m*/*z* 551. It yielded the *m*/*z* 457 ion resulting from the neutral loss of phenol on flavonoid part II, ^0,2^IIA^−^ ion at *m*/*z* 431, ^0,2^IIA^−^ − H_2_O ion at *m*/*z* 413, and the ^0,4^IIA^−^ ion at *m*/*z* 389 in the MS/MS spectrum, 14 Da higher than those of amentoflavone, as well as the [M − H − C_6_H_6_O − CH_3_•]^−^ ion at *m*/*z* 442, and the ^0,4^IIB^−^ (^1,3^IA^−^) ion at *m*/*z* 151, indicating that peak 6 could be a methylated amentoflavone, and the methoxyl group could be linked to C4′ or C7′′. Although the ion from the C–ring cleavage of flavonoid part I at position 1/3 was not found in the MS/MS spectrum of amentoflavone in our experiment, the fragment was observed in the positive ion mode for amentoflavone-type biflavones with relatively low abundance [24]. After a literature research, peak 6 was tentatively identified as bilobetin (4′-*O*-methylamentoflavone) [23], whose proposed fragmentation pathway is shown in Figure S4 in the Supplementary Materials.

Peak 7 at t_R_ 31.6 min gave an [M − H]^−^ ion at *m*/*z* 521, 14 Da lower than that of amentoflavone. It yielded the ^0,4^IIA^−^ ion at *m*/*z* 375 corresponding to the base peak, and the ^0,4^IIA^−^ − CO_2_ ion at *m*/*z* 331 in the MS^2^ spectrum, and the peak abundance of these two product ions were identical to that of amentoflavone. Hence, peak 7 could be potentially identified as 4′′′-dehydroxyamentoflavone, which has been identified from *S. doederleinii* for the first time.

The [M − H]^−^ ion at *m*/*z* 565 of peak 14 at t_R_ 39.7 min produced predominantly the [M − H − CH_3_OH]^−^ ion at *m*/*z* 533 in the MS/MS spectrum, suggesting a methoxyl group could be linked to C4′ or C7′′. The ^0,4^IIA^−^ ion at *m*/*z* 389 was identical to that of peak 6, indicating that another methoxyl group could be linked to C4′′′. In addition, the *m*/*z* 518, 507, 415, 388, 374, and 151 ions could be attributed to the [M − H − CH_3_OH − CH_3_•]^−^, [M − H − C_3_H_6_O]^−^, ^1,3^IIA^−^ − H_2_O, ^0,4^IIA^•−^, ^0,4^IIA^−^ − CH_3_•, and ^0,4^IIB^−^ (^1,3^IA^−^) ions, respectively. Thus, peak 14 was preliminarily characterized as isoginkgetin (4′,4′′′-dimethylamentoflavone). The proposed fragmentation pathway of isoginkgetinis shown in Figure S5 in the Supplementary Materials.

The [M − H]^−^ ion at *m*/*z* 579 of peak 17 at t_R_ 46.5 min produced the [M − H − C_2_H_5_OH]^−^ ion at *m*/*z* 533 and [M − H − CH_3_OH]^−^ ion at *m*/*z* 547 in the MS/MS spectrum, suggesting that two methoxyl groups could be linked to C4′ and C7′′. Besides, the ^0,4^IIA^−^ ion at *m*/*z* 403 and ^1,3^IIA^−^ − CH_3_OH ion at *m*/*z* 415 have indicated that another methoxyl group could be linked to C4′′′. In addition, the *m*/*z* 388 ion could be attributed to ^0,4^IIA^−^ − CH_3_•. Thus, peak 17 was preliminarily characterized as kayaflavone (4′,7′′,4′′′-trimethylamentoflavone) [26], which has been identified from *S. doederleinii* for the first time. The proposed fragmentation pathway of kayaflavoneis shown in Figure S6 in the Supplementary Materials.

Peak 19 at t_R_ 49.6 min generated the [M − H]^−^ ion at *m*/*z* 581, which yielded the ^1,3^IA^−^ product ion at *m*/*z* 165, suggesting a methoxyl group could substitute on ring A of flavone part I. In addition, the ^0,4^IIA^−^ion at *m*/*z* 403 (base peak) and the ^1,3^IB^−^ − CH_3_OH ion at *m*/*z* 383 in the MS/MS spectrum indicated that a methoxyl group could be linked to C4′ or C7′′ and the third methoxyl group could be linked to C4′′′. Thus, peak 19 was tentatively assigned as 2′′,3′′-dihydroheveaflavone, which has been identified from *S. doederleinii* for the first time.

The [M − H]^−^ ion at *m*/*z* 537 of peak 3 at 22.8 min produced product ions at *m*/*z* 519, 385, 375 and 151 in the MS^2^ spectrum. Since there were no diagnostic ions at *m*/*z* 269 and 284 that resulted from the rupture of the connective C–O bond, peak 3 could be classified to C–C linked biapigenin, and the ions at *m*/*z* 385, 375, and 151 could correspond to ^1,3^IB^−^, ^0,4^IIA^−^ , and ^1,3^IA^−^ ions, respectively. After a literature research, peak 3 was tentatively identified as 2′,8′′-biapigenin [30], and the [M − H_2_O]^−^ ion at *m*/*z* 519 also supports this. The proposed fragmentation pathway is shown in Figure S7 in the Supplementary Materials.

#### 2.2.2. Characterization of IC3′–IIC6′′ Linked Biflavonoids from *S. doederleinii*

Peak 15 with a t_R_ of 40.5 min gave a deprotonated molecule [M − H]^−^ ion at *m*/*z* 565. In the product ion mass spectrum of the [M − H]^−^ ion, a series of prominent ions at *m*/*z* 445 (base peak), 403, 427 and 117 were observed, corresponding to the ^0,2^IIA^−^, ^0,4^IIA^−^, ^1,4^IB^−^, and ^1,3^IIB^−^ fragments of the robustflavone standard. Peak 15 is clearly a robustflavone-type biflavonoid because of the typical retrocyclization fragmentation involving the 1 and 4 bonds on flavonoid part I. Other product ions, such as *m*/*z* 430 (^0,2^IIA^−^ − CH_3_•), 412 (^1,4^IB^−^ − CH_3_•), 388 (^0,4^IIA^−^ − CH_3_•), and 372 (^0,4^IIA^−^ − OCH_3_•) also support this, indicating that this molecule has two methoxyl groups, one of them on ring A of flavone part I, and no methoxyl group on ring B of flavone part II. Additionally, the *m*/*z* 533, 471, and 456 ions corresponded to the [M − H − CH_3_OH]^−^, [M − H − C_6_H_6_O]^−^, [M − H − C_6_H_6_O − CH_3_•]^−^ ions, respectively. Thus, peak 15 was preliminarily characterized as robustaflavone 7,4′-dimethyl ether from the *Selaginella* genus [27], which has been identified from *S. doederleinii* for the first time. The proposed fragmentation pathway is shown in Figure S8 in the Supplementary Materials.

#### 2.2.3. Characterization of IC3′–IIC3′′′ Linked Biflavonoids from *S. doederleinii*

Peak 12 at t_R_ 37.2 min showed the [M − H]^−^ ion at *m*/*z* 553, 14 Da higher than that of 2′′,3′′-dihydro-3′,3′′′-biapigenin. Additionally, these two compounds have identical product ions at *m*/*z* 413, 387, 369, and 151 in the MS/MS spectra. Hence, peak 12 could be preliminarily characterized as 2′′,3′′-dihydro-3′,3′′′-biapigenin methyl ether, which has been identified from *S. doederleinii* for the first time.

Peak 13, detected at t_R_ 37.8 min showed the [M − H]^−^ ion at *m*/*z* 555, 14 Da higher than that of 3′,3′′′-binaringen. These two compounds yield identical product ions at *m*/*z* 237, 151, 263, and 219 in the MS/MS spectra, attributed to the ^1,3^IB^1,3^IIB^−^, ^1,3^IA^−^ (^1,3^IIA^−^), ^1,3^IB^0,4^IIB^−^ − H_2_O (^0,4^IB^1,3^IIB^−^ − H_2_O), and ^1,3^IB^1,3^IIB^−^ − H_2_O ions, respectively. Furthermore, the *m*/*z* 403, 165, and 429 product ions of peak 13 correspond to the ^1,3^IB^−^(^1,3^IIB^−^), ^1,3^IA^−^ (^1,3^IIA^−^), and ^0,4^IB^−^ − H_2_O (^0,4^IIB^−^ − H_2_O) fragments of the 3′,3′′′-binaringen standard, and are 14 Da higher than those of 3′,3′′′-binaringen, indicating that peak 13 could be a 3′,3′′′-binaringen methyl ether, and the methylated group is substituted on ring A of either flavanone I or II. Hence, peak 13 could be preliminarily characterized as 3′,3′′′-binaringen methyl ether, which has been identified from *S. doederleinii* for the first time. The proposed fragmentation pathway is shown in Figure S9 in the Supplementary Materials.

#### 2.2.4. Characterization of C–O Linked Biflavonoids from *S. doederleinii*

Peak 9 at t_R_ 33.6 min gave an [M − H]^−^ ion at *m*/*z* 537. The most main diagnostic ions in the MS/MS spectrum are a sequence of ions at *m*/*z* 285, 284, 269, and 256, which result from the rupture of the C–O connection on flavonoid parts I and II. Other product ions at *m*/*z* 493, 469, 385 and 151 were attributed to the [M − H − CO_2_]^−^, [M − H − C_3_O_2_]^−^, ^1,3^IB^−^ and ^1,3^IA^−^ ions, respectively. Hence, peak 9 could be tentatively characterized as a known hinokiflavone from this herb [23]. The proposed fragmentation pathway is shown in Figure S10 in the Supplementary Materials.

Peak 10 at t_R_ 34.2 min gave an [M − H]^−^ ion at *m*/*z* 539. The product ions at *m*/*z* 495, 471, 455, 453, 411, 387, 284, 256, and 151 in the MS/MS spectrum were identical to those of chrysocauloflavone I, suggesting that peak 10 could be a C–O linked biflavonoid, and its two flavonoid parts were similar with chrysocauloflavone I. In addition, the *m*/*z* 191 ion could be attributed to the [M − H]^−^ ion of 2,3-dehydro-5,6,7-trihydroxy-chromen-4-one, indicating that there were potentially three hydroxy groups on ring A. Thus, after a literature search, peak 10 could be tentatively characterized as a known 2,3-dihydrohinokiflavone from the *Selaginella* genus [28], which has been identified from *S. doederleinii* for the first time. The pathway proposed to explain the fragmentations is shown in Figure S11 in the Supplementary Materials.

The [M − H]^−^ ion at *m*/*z* 553 of peak 16 at t_R_ 43.4 min yielded a sequence of ions at *m*/*z* 299, 298, 284, 283, 255, and 165 in the MS/MS spectrum, which result from the rupture of the C–O connection on flavonoid parts I and II. Furthermore, the *m*/*z* 509, 485, 469, 467, 425, and 401 product ions of peak 16 correspond to the [M − H − CO_2_]^−^, [M − H − C_3_O_2_]^−^, [M − H − C_4_H_4_O_2_]^−^, [M − H − C_2_H_2_O − CO_2_]^−^, [M − H − C_4_H_4_O_2_ − CO_2_]^−^, and ^1,3^IB^−^ fragments of peak 10, and are 14 Da higher than those of peak 10, indicating that peak 16 could be a 2,3-dihydrohinokiflavone methyl ether, and the methylated group is substituted on ring A of flavanone II. Other product ions, such as *m*/*z* 386 (^1,3^IB^−^ − CH_3_•), 151 (^1,3^IA^−^) and 125 (^1,4^IA^−^) also support this. After a thorough literature search, peak 16 was preliminarily characterized as a known 2,3-dihydroisocryptomerin from the *Selaginella* genus [29,31], which has been identified from *S. doederleinii* for the first time. The proposed fragmentation pathway is shown in Figure S12 in the Supplementary Materials.

### 2.3. In Vitro and In Vivo Anticancer Activity of the Total Biflavonoids Extract

**Figure 6:**
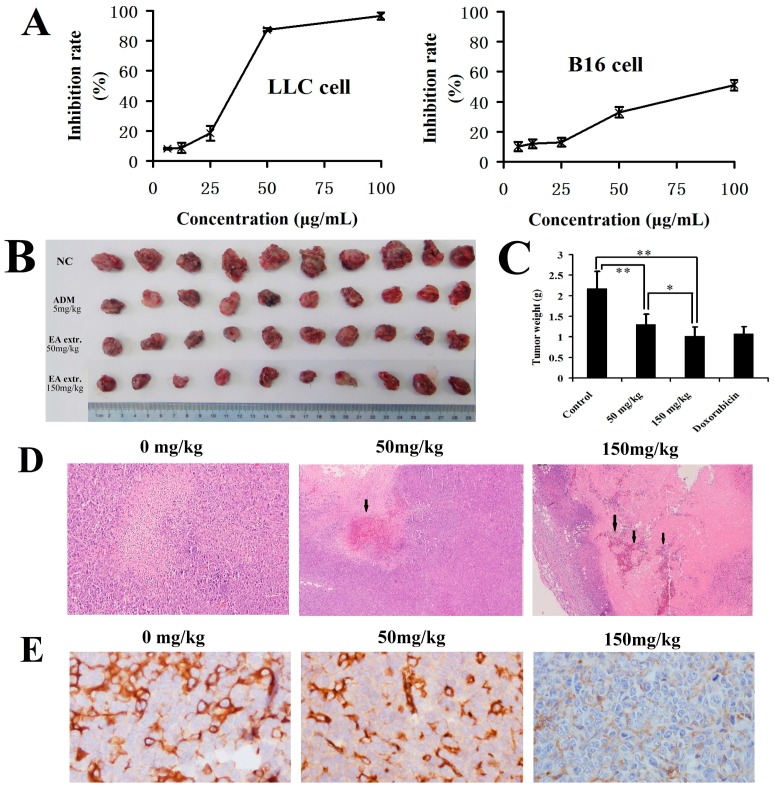
Effect of the biflavonoids extract of *S. doederleinii* on (**A**) LLC and B16 cells growth; and (**B**) tumor growth in male C57BL/6 mice. Mice were inoculated with LLC for 2 weeks prior to per os (p.o.) 50 and 150 mg/kg of the extract dissolved in component solvent (consisting of double distilled water, ethanol, propylene glycol, and PEG-400) once a day for 12 days; (**C**) Tumor weights were recorded. Data are expressed as means ± SD, *n* = 10 mice per group. * *p* < 0.05; ** *p* < 0.01, significantly different as indicated; (**D**) Hematoxylin and eosin (H&E) staining (×100), and (**E**) CD31 staining (×400) of xenograft tumors in each group. Arrows indicate the tumor necrosis region.

According to the results from the HPLC-ESI-QTOF-MS analysis, it was confirmed that the ethyl acetate extract of *S. doederleinii* mainly consisted of biflavonoids. In order to study the effect of the total biflavonoids extract on cancer cell growth, LLC and B16 cells were treated with various doses of the extract for 48 h, and the viabilities were then determined by the MTT assay. The results showed that the total biflavonoids extract dose-dependently decreased the viability of the LLC and B16 cells (Figure 6A) with mean IC_50_ values of 36.29 and 95.65 μg/mL, respectively.

To further validate the anti-cancer effect of the extract, LLC xenograft-bearing mice were administered the extract p.o. at a dose of 50 or 150 mg/kg/day. As shown in Figure 6B,C, the total biflavonoids extract inhibited tumor growth in the xenograft models by 40.11% and 53.5% for low-dose and high-dose groups, respectively, without the loss of body weight (Table S2 in the Supplementary Materials). By H&E staining examination (Figure 6D), it was shown that the low-dose and high-dose total biflavonoids caused tumor necrosis. As shown in Figure 6E and Table S3 in the Supplementary Materials, both the 50 and 150 mg/kg doses of the extract significantly decreased the MVD count of xenograft tumors (*p* <0.01, vs. model group), respectively. Therefore, the total biflavonoids extract obviously reduced the microvessel density of xenograft tumors in a dose-dependent manner.

In addition, as shown in Table S4 in the Supplementary Materials, the expression levels of serum TNF-α and IFN-γ of the high dose group were significantly higher than the other three groups (TNF-α: *p* < 0.01, high dose group vs. Model group, high-dose group vs. ADM group, high-dose group vs. low-dose group; IFN-γ: *p* < 0.01, high dose group vs. model group, high-dose group vs. low-dose group). The TNF-α and IFN-γ levels of the low-dose group were also significantly higher than those of the model group (*p* < 0.05). The results suggested that the immune response was enhanced by the total biflavonoids in a dose-dependent manner, which could be one of the antitumor mechanisms for the total biflavonoids.

## 3. Experimental

### 3.1. Reagents and Materials

Methanol and acetonitrile (HPLC-grade) were purchased from Merck (Darmstadt, Germany) while acetic acid glacial (HPLC-grade) was purchased from Aladdin (Shanghai, China). Ethanol was obtained from Sinopharm Chemical Reagents (Shanghai, China). Double distilled water was used for all the preparations. Amentoflavone, robustaflavone, 2′′,3′′-dihydro-3′,3′′′-biapigenin,3′,3′′′-binaringenin, delicaflavone, chrysocauloflavone I, heveaflavone, and 7,4′,7′′,4′′′-tetra-*O*-methyl-amentoflavone were separated from *S. doederleinii* and their structures were elucidated by MS, UV, ^1^H-NMR, and ^13^C-NMR and confirmed by comparison with the literature values [2,4,6]. The purity of the compounds was higher than 98% according to HPLC analysis by peak area normalization. All the solutions were filtered through a 0.45 μm membrane and degassed by ultrasonic bath before being submitted to HPLC-(−)ESI-QTOF MS/MS analysis.

The plant materials of *S. doederleinii* were purchased from a local Chinese medicine store in Fuzhou (China) and authenticated by associate professor Hong Yao (Department of Pharmaceutical Analysis, Fujian Medical University, Fuzhou, Fujian, China). The voucher specimens (No. 120405) were deposited in the phytochemistry laboratory, Fujian Medical University, Fujian, China.

### 3.2. Standard Solutions and Sample Preparation

Methanolic stock solutions containing biflavonoid reference compounds with known concentrations (about 20 μg/mL) were prepared and stored in a refrigerator (4 °C) until they were required for use. The extract was prepared according to the protocols in the literature [8,10] with slight modification. Described briefly, the dried whole plants of *S. doederleinii* were pulverized into powder (20–40 mesh). The sample powder was extracted at 85 °C for 2 h with an 8-fold volume of 70% ethanol three times, and the resulting ethanol extracts were combined, transferred to a flask, and evaporated in a rotatory evaporator (Buchi, Switzerland) to condense into a paste. The ethanol extract was extracted twice with petroleum ether and was then filtered. The residues were extracted twice with an 8-fold volume of dichloromethane and were then filtered. The residues were then extracted twice with an 8-fold volume of ethyl acetate and were filtered. Ultimately, the ethyl acetate filtrate was concentrated using a rotary evaporator at 55 °C, and it was lyophilized to obtain the total biflavones extract (a powder).

For HPLC-ESI-QTOF MS/MS analysis, an aliquot (about 1 g) was accurately weighed and ultrasonically extracted with 50 mL of 70% aqueous methanol for 1 h. The extract was filtered through a 0.45 μm membrane and 10 μL aliquots were injected into the HPLC.

### 3.3. HPLC-ESI-QTOF MS/MS Analysis

HPLC analysis was applied on an Agilent 1290 Infinity LC instrument (Agilent, Waldbronn, Germany) consisting of a binary pump, a diode-array detector, an auto-sampler, and a column compartment. The samples were separated on an Ultimate ^TM^XB-C_18_ column, 5 μm, 250 mm × 4.6 mm i.d. (Welch Materials, Inc., Ellicott, MD, USA) and on-line UV spectra were recorded in the wavelength range from 200–400 nm. The mobile phase was a stepwise gradient of water (containing 0.5% acetic acid, *v*/*v*) and acetonitrile (0 min, 65:35; 4 min, 56:44; 23 min, 54:46; 25 min, 48:52; 38 min, 26:74; 40 min, 25.5:74.5; 46 min, 16:84; 50–68 min, 0:100). The column temperature was set at 30 °C and the flow rate was 0.5 mL/min.

The HPLC system was connected to an Agilent 6530 QTOF mass spectrometer (Santa Clara, CA, USA) equipped with an ESI interface using the following operation parameters: capillary voltage, 3.5 kV ((−)ESI mode); nebuliser, 45 psig; drying gas (nitrogen) flow rate, 8.0 L/min; sheath gas flow rate, 10.0 L/min; gas temperature, 350 °C; fragmentor, 175 V; skimmer voltage, 65 V; OCT 1 RF Vpp, 750 V. Mass spectra were recorded across the range *m*/*z* 100–1500, and all masses were corrected by the internal standards provided by Agilent Technologies (Agilent Part Number: G1969-85001) with *m*/*z* at 112.98559 and 1033.98811 in (−)ESI mode. The collision energy for collision-induced dissociation (CID) in the MS/MS spectra in (−)ESI mode was adjusted to 35% of the maximum. The data of accurate mass, mass error, molecular weight (MW), and chemical formula were processed with Agilent Mass Hunter Workstation Software version B.06.00 (Agilent Technologies).

### 3.4. Cell Lines and Culture

Mouse lung cancer LLC cell and B16 melanoma cell lines were obtained from the Type Culture Collection of the Chinese Academy of Sciences (Shanghai, China) and maintained in a humidified incubator containing 5% CO_2_ at 37 °C. A549 cells were cultured in DMEM/high glucose medium (HyClone, Logan, UT, USA) supplemented with 10% fetal bovine serum (FBS; Hangzhou Evergreen Biological Engineering Materials Co., Ltd., Hangzhou, China), and 1% penicillin–streptomycin. Cells were grown in plastic tissue culture dishes and harvested with a solution of trypsin–EDTA while in a logarithmic phase of growth. All the experiments were performed on logarithmically growing cells.

### 3.5. Cell Viability Assay

The effect of the total biflavonoids extract on cell growth was evaluated by a previous method with slight modification [6]. Described briefly, 3 × 10^3^ cells were seeded in 96-well plates and permitted to incubate overnight. After exposure to the designated doses of the total biflavonoids extract for 48 h, 20 µL of MTT solution (5 mg/mL in PBS) were added to each well of the 96-well plates. The plates were incubated for 4 h at 37 °C. After that, the media in the plates were removed and 150 µL DMSO was added to each well to solubilize the formazan crystals. The absorbance was determined using a 96-well microplate reader at 490 nm. The total biflavonoids extract was dissolved in DMSO and further diluted in medium. The final DMSO concentration was 0.1%.

### 3.6. In Vivo Anticancer Test

The animal care and experimental protocols were approved by the Animal Care and Ethics Committee, Fujian Medical University (Fuzhou, China). All mice were housed in controlled conditions (temperature: 22 ± 2 °C and lighting: 8:00–20:00) and received a standard mouse chow and tap water adlibitum.

The in vivo anticancer effect of the total biflavonoids extract was evaluated according to previous method with slight modification [10,11]. Described briefly, the mouse xenograft model was established by injection of 2 × 10^6^ LLC cells s.c. into the right armpit of six to eight-week old C57BL/6 male mice (18–22 g, obtained from the National Rodent Laboratory Animal Resource, Shanghai, China). The mice were randomized into vehicle control, positive, and treatment groups of ten animals when xenografts were palpable with an average size of 50–70 mm^3^. Vehicle (Solvent) or treatment groups (50 and 150 mg/kg extracts) were administered p.o. every day for 12 days. The positive group was administered i.v. 5 mg/kg Doxorubicin (ADM) once every three days via the tail vein until sacrifice. Body weight was measured and recorded every two days. The day after the last treatment, blood was collected from the orbital sinus in the mice, and the mice were then sacrificed by cervical dislocation and the tumors were collected, weighed, and photographed. The blood samples were placed in a 4 °C refrigerator for 4 h, followed by centrifugation at 1000× *g* for 20 min. The resultant serum samples were stored at −20 °C before use. The tumor inhibition effect was calculated using the following equation: tumor suppression (%) = (1 − T/C) × 100. T is the average tumor weight of the treated group and C is that of the control group.

### 3.7. Immunohistochemistry and Microvessel Density (MVD) Assessment

Tumors were fixed in 10% neutral formalin for 24 h. The tissue was processed for sectioning and staining by standard histological methods. Some sections (5 μm) were stained with hematoxylin and eosin and observed under inverted light microscopy (BX41; Olympus Optical Co., Tokyo, Japan) at 100× magnification. For MVD assessment, other sections (5 μm) were co-cultured with goat anti-mouse monoclonal CD31 antibody. For the quantification of positively stained vessels, the number of microvessels was counted in seven randomly chosen high-power fields by a pathology doctor at 400× magnification.

### 3.8. Determination of TNF-α and IFN-γ in Mice Serum

TNF-α and IFN-γ in mice serum were measured using TNF-α and IFN-γ ELISA kits (Boster Biological Technology, Ltd., Wuhan, China), according to the manufacturer’s instructions*.*

### 3.9. Data Analysis

Data were expressed as means ± SD. A one-way analysis of variance (ANOVA) was used to compare the means among different groups with SPSS18.0 software. A *p* value < 0.05 was considered to be statistically significant.

## 4. Conclusions

In this paper, the fragmentation patterns in (−)ESI mode of biflavonoids from the *Selaginella* genus, including IC3′–IIC8′′, IC3′–IIC6′′, IC3′–IIC3′′′, and C–O linked biflavonoids were proposed in detail using QTOF MS. For the IC3′–IIC8′′ linked biflavonoids, the most useful fragmentation in (−)ESI mode involves cleavage of the C–ring of flavonoid part II at position 0/4. For the IC3′–IIC6′′ linked biflavonoids, the retrocyclization fragmentation involving 1 and 4 bonds on flavonoid part I and leading to the ^1,4^IB^−^ ion in the MS^2^ spectrum could be used to distinguish from the IC3′–IIC8′′ linked biflavonoids. The predominant diagnostic ions of the C–O linked biflavonoids were flavonoid aglycone related ions that resulted from the rupture of the connective C–O bond. In addition, the fragmentation behaviors of the IC3′–IIC3′′′ linked biflavonoids were investigated for the first time. Fragmentation routes involving the cleavage of the C–ring at positions 1/3 and 0/4 and leading to the ^1,3^IB^−^ (^1,3^IIB^−^) and ^0,4^IB^−^ (^0,4^IIB^−^) ions are the main pathways of the IC3′–IIC3′′′ linked biflavonoids. Furthermore, the cleavage at positions 1/3 and 0/4 occurred more easily in the flavanone part than in the flavone part, producing characteristic product ions with small molecular weights, such as the ^1,3^IB^0,4^IIB^−^ − H_2_O (^0,4^IB^1,3^IIB^−^ − H_2_O) ion at *m*/*z* 263, ^1,3^IB^1,3^IIB^−^ ion at *m*/*z* 237, and ^1,3^IB^1,3^IIB^−^ − H_2_O ion at *m*/*z* 219. Combined with HPLC, the established approach for the structural identification of biflavonoids by QTOF MS was applied to the analysis of the *S. doederleinii* extract. A total of twenty biflavonoids were identified or tentatively characterized, including eight IC3′–IIC8′′, one I2′–IIC8′′, two IC3′–IIC6′′, four IC3′–II3′′′, and five C–O linked biflavonoids. Among them, eight biflavonoids were identified from *S. doederleinii* for the first time. The results of this work could serve as an effective tool for the identification of biflavonoids in herbal samples.

Furthermore, we validated the in vitro and in vivo anticancer activity of the total biflavonoids extract from *S. doederleinii* by the MTT assay and a xenograft model of mouse lung cancer LLC in male C57BL/6 mice. The total biflavonoids extract enhanced the antitumor immune response in the mouse lung cancer model.

In summary, the presented work could provide useful knowledge for the identification of biflavonoids in herbal samples and further insights into the chemopreventive function of this plant.

## Figures and Tables

**Table 1:** Peak assignments for the analysis of the extract from *S. doederleinii*.

Peak No.	t_R_ (min)	Identification	(−)ESI–MS *m*/*z*		Formula
			Observed	Calculated (Δppm)	
1	17.4	Amentoflavone	537.082	537.0827 (−1.30)	C_30_H_18_O_10_
2	19.6	Robustaflavone	537.0815	537.0827 (−2.23)	C_30_H_18_O_10_
3	22.8	2′,8′′-Biapigenin	537.0824	537.0827 (−0.56)	C_30_H_18_O_10_
4	25.6	2′′,3′′-Dihydro-3′, 3′′′-biapigenin	539.0974	539.0984 (−1.85)	C_30_H_20_O_10_
5	27.9	3′,3′′′-Binaringenin	541.113	541.114 (−1.85)	C_30_H_22_O_10_
6	28.9	Bilobetin	551.0977	551.0984 (−1.27)	C_31_H_20_O_10_
7	31.6	4′′′-Dehydroxyamentoflavone ^a^	521.0871	521.0878 (−1.34)	C_30_H_18_O_9_
8	33.0	Delicaflavone	537.0822	537.0827 (−0.93)	C_30_H_18_O_10_
9	33.6	Hinokiflavone	537.0819	537.0827 (−1.49)	C_30_H_18_O_10_
10	34.2	2,3-Dihydrohinokiflavone ^a^	539.0974	539.0984 (−1.85)	C_30_H_20_O_10_
11	34.8	Chrysocauloflavone I	539.0976	539.0984 (−1.48)	C_30_H_20_O_10_
12	37.2	2′′,3′′-Dihydro-3′,3′′′-biapigenin methyl ether ^a^	553.1134	553.114 (−1.08)	C_31_H_22_O_10_
13	37.8	3′,3′′′-Binaringenin methyl ether ^a^	555.1285	555.1297 (−2.16)	C_31_H_24_O_10_
14	39.7	Isoginkgetin	565.1133	565.114 (−1.24)	C_32_H_22_O_10_
15	40.5	Robustaflavone 7,4′-dimethyl ether ^a^	565.1135	565.114 (−0.88)	C_32_H_22_O_10_
16	43.4	2,3-Dihydroisocryptomerin ^a^	553.1132	553.114 (−1.45)	C_31_H_22_O_10_
17	46.5	4′,7′′,4′′′-Trimethylamentoflavone ^a^	579.1289	579.1297 (−1.38)	C_33_H_24_O_10_
18	47.7	Heveaflavone	579.1286	579.1297 (−1.90)	C_33_H_24_O_10_
19	49.6	2′′,3′′-Dihydroheveaflavone ^a^	581.1435	581.1453 (−3.10)	C_33_H_26_O_10_
20	54.1	7,4′,7′′,4′′′-Tetra-*O*-methyl-amentoflavone	593.1446	593.1453 (−1.18)	C_34_H_26_O_10_

^a^ New compounds from *S. doederleinii*.

**Table 2:** Q-TOF MS/MS data obtained in (−)ESI mode of biflavonoids of *S. doederleinii*.

	(−)ESI-MS^2^ *m*/*z* (% Base Peak)				(−)ESI-MS^2^ *m*/*z* (% Base Peak)		
Peak	Observed Mass	Calculated Mass (Δppm)	Proposed Formula	Peak	Observed Mass	Calculated Mass (Δppm)	Proposed Formula
**IC3′ (I2′)–IIC8′′Linked Biflavonoids**							
3	MS^2^[537]:			7	MS^2^[521]:		
	537.0824 (100)	537.0827 (−0.56)	C_30_H_17_O_10_^−^		375.05 (100)	375.051 (−2.67)	C_21_H_11_O_7_^−^
	385.071 (25)	385.0718 (−2.08)	C_23_H_13_O_6_^−^		331.0595 (15)	331.0612 (−5.14)	C_20_H_11_O_5_^−^
	519.0715 (14)	519.0722 (−1.35)	C_30_H_15_O_9_^−^		521.0871 (6)	521.0878 (−1.34)	C_30_H_17_O_9_^−^
	151.0028 (10)	151.0037 (−5.96)	C_7_H_3_O_4_^−^	14	MS^2^[565]:		
	375.05 (7)	375.051 (−2.67)	C_21_H_11_O_7_^−^		533.0868 (100)	533.0878 (−1.88)	C_31_H_17_O_9_^−^
6	MS^2^[551]:				518.063 (22)	518.0643 (−2.51)	C_30_H_14_O_9_^−•^
	457.0586 (100)	457.0565 (4.59)	C_25_H_13_O_9_^−^		507.0713 (12)	507.0722 (−1.77)	C_29_H_15_O_9_^−^
	431.0793 (93)	431.0772 (4.87)	C_24_H_15_O_8_^−^		565.1133 (10)	565.114 (−1.24)	C_32_H_21_O_10_^−^
	389.0688 (64)	389.0667 (5.40)	C_22_H_13_O_7_^−^		389.0654 (10)	389.0667 (−3.34)	C_22_H_13_O_7_^−^
	151.0036 (27)	151.0037 (−0.66)	C_7_H_3_O_4_^−^		415.0445 (6)	415.0459 (−3.37)	C_23_H_11_O_8_^−^
	442.0342 (25)	442.0330 (2.71)	C_24_H_10_O_9_^−•^		374.0427 (5)	374.0432 (−1.34)	C_21_H_10_O_7_^−•^
	413.0689 (23)	413.0667 (5.33)	C_24_H_13_O_7_^−^		388.0577 (5)	388.0589 (−3.09)	C_22_H_12_O_7_^−•^
	551.0977 (18)	551.0984 (−1.27)	C_31_H_19_O_10_^−^		151.003 (4)	151.0037 (−4.64)	C_7_H_3_O_4_^−^
17	MS^2^[579]:			19	MS^2^[581]:		
	533.086 (100)	533.0878 (−3.38)	C_31_H_17_O_9_^−^		403.0836 (100)	403.0823 (−1.49)	C_23_H_15_O_7_^−^
	579.1289 (42)	579.1297 (−1.38)	C_33_H_23_O_10_^−^		581.1435 (53)	581.1453 (−3.10)	C_33_H_25_O_10_^−^
	388.0573 (7)	388.0589 (−4.12)	C_22_H_12_O_7_^−•^		165.0193 (46)	165.0193 (0)	C_8_H_5_O_4_^−^
	403.0817 (6)	403.0823 (−1.49)	C_23_H_15_O_7_^−^		383.0934 (40)	383.0925 (2.35)	C_24_H_15_O_5_^−^
	547.101 (4)	547.1035 (−4.57)	C_32_H_19_O_9_^−^				
	415.0472 (2)	415.0459 (3.13)	C_23_H_11_O_8_^−^				
**IC3′–IIC6′′Linked Biflavonoid**							
15	MS^2^[565]:						
	445.0919 (100)	445.0929 (−2.25)	C_25_H_17_O_8_^−^		430.0683 (36)	430.0694 (−2.56)	C_24_H_14_O_8_^−•^
	388.058 (86)	388.0589 (−2.32)	C_22_H_12_O_7_^−•^		372.0629 (29)	372.0639 (−2.69)	C_22_H_12_O_6_^−•^
	403.0813 (80)	403.0823 (−2.48)	C_23_H_15_O_7_^−^		412.0575 (27)	412.0589 (−3.40)	C_24_H_12_O_7_^−•^
	456.0477 (50)	456.0487 (−2.19)	C_25_H_12_O_9_^−•^		117.0343 (23)	117.0346 (−2.56)	C_8_H_5_O^−^
	471.0709 (43)	471.0722 (−2.76)	C_26_H_15_O_9_^−^		533.0854 (16)	533.0878 (−4.50)	C_31_H_17_O_9_^−^
	427.0812 (42)	427.0823 (−2.58)	C_25_H_15_O_7_^−^				
**IC3′–IIC3′′′Linked Biflavonoids**							
12	MS^2^[553]:			13	MS^2^[555]:		
	387.0869 (100)	387.0874 (−1.29)	C_23_H_15_O_6_^−^		237.0921 (100)	237.0921 (0)	C_16_H_13_O_2_^−^
	369.0763 (8)	369.0768 (−1.35)	C_23_H_13_O_5_^−^		151.0036 (14)	151.0037 (−0.66)	C_7_H_3_O_4_^−^
	413.0657 (2)	413.0667 (−2.42)	C_24_H_13_O_7_^−^		403.1174 (13)	403.1187 (−3.22)	C_24_H_19_O_6_^−^
	151.0032 (2)	151.0037 (−3.31)	C_7_H_3_O_4_^−^		263.0711 (10)	263.0714 (−1.14)	C_17_H_11_O_3_^−^
					165.0187 (5)	165.0193 (−3.64)	C_8_H_5_O_4_^−^
					219.0808 (4)	219.0815 (−3.20)	C_16_H_11_O^−^
					429.0969 (3)	429.098 (−2.56)	C_25_H_17_O_7_^−^
**C–O Linked Biflavonoids**							
9	MS^2^[537]:			16	MS^2^[553]:		
	537.0819 (100)	537.0827 (−1.49)	C_30_H_17_O_10_^−^		401.102 (100)	401.1031 (−2.74)	C_24_H_17_O_6_^−^
	284.0318 (14)	284.0326 (−2.82)	C_15_H_8_O_6_^−•^		469.0931 (29)	469.0929 (0.43)	C_27_H_17_O_8_^−^
	269.0443 (11)	269.0455 (−4.46)	C_15_H_9_O_5_^−^		225.0063 (27)	225.0041 (9.78)	C_9_H_5_O_7_^−^
	151.0034 (10)	151.0037 (−1.99)	C_7_H_3_O_4_^−^		467.1128 (26)	467.1136 (−1.71)	C_28_H_19_O_7_^−^
	285.0392 (10)	285.0405 (−4.56)	C_15_H_19_O_6_^−^		299.0534 (24)	299.0561 (−9.03)	C_16_H_11_O_6_^−^
	469.0918 (9)	469.0929 (−2.34)	C_27_H_17_O_8_^−^		553.1132 (23)	553.114 (−1.45)	C_31_H_21_O_10_^−^
	385.0709 (8)	385.0718 (−2.34)	C_23_H_13_O_6_^−^		509.1231 (21)	509.1242 (−2.16)	C_30_H_21_O_8_^−^
	256.0365 (7)	256.0377 (−4.69)	C_14_H_8_O_5_^−•^		386.078 (19)	386.0796 (−4.14)	C_23_H_14_O_6_^−•^
	493.0920 (5)	493.0929 (−1.83)	C_29_H_17_O_8_^−^		298.047 (15)	298.0483 (−4.36)	C_16_H_10_O_6_^−•^
10	MS^2^[539]:				151.0035 (11)	151.0037 (−1.32)	C_7_H_3_O_4_^−^
	495.1081 (100)	495.1085 (−0.81)	C_29_H_19_O_8_^−^		164.9846 (11)	164.9829 (10.30)	C_7_HO_5_^−^
	453.0973 (27)	453.0980 (−1.54)	C_27_H_17_O_7_^−^		425.1014 (10)	425.1031 (−4.00)	C_26_H_17_O_6_^−^
	284.0325 (26)	284.0326 (−0.35)	C_15_H_8_O_6_^−•^		284.0315 (9)	284.0326 (−3.87)	C_15_H_8_O_6_^−•^
	387.0869 (22)	387.0874 (−1.29)	C_23_H_15_O_6_^−^		255.0296 (7)	255.0299 (−1.18)	C_14_H_7_O_5_^−^
	151.0035 (15)	151.0037 (−1.32)	C_7_H_3_O_4_^−^		283.0261 (6)	283.0248 (4.59)	C_15_H_7_O_6_^−^
	455.0784 (14)	455.0772 (2.64)	C_26_H_15_O_8_^−^		485.1225 (6)	485.1242 (−3.50)	C_28_H_21_O_8_^−^
	539.0974 (14)	539.0984 (−1.85)	C_30_H_19_O_10_^−^		125.0239 (5)	125.0244 (−4.00)	C_6_H_5_O_3_^−^
	190.9985 (13)	190.9986 (−0.52)	C_9_H_3_O_5_^−^				
	256.0371 (10)	256.0377 (−2.34)	C_14_H_8_O_5_^−•^				
	255.0298 (10)	255.0299 (−0.39)	C_14_H_7_O_5_^−^				
	411.0864 (9)	411.0874 (−2.43)	C_25_H_15_O_6_^−^				
	471.108 (9)	471.1085 (−1.06)	C_27_H_19_O_8_^−^				
	268.037 (7)	268.0377 (−2.61)	C_15_H_8_O_5_^−•^				

## Supplementary Materials

The following are available online.
