# Supplementary material for: Analysis of the Total Biflavonoids Extract from Selaginella doederleinii by HPLC-QTOF-MS and Its In Vitro and In Vivo Anticancer Effects
Source: Molecules. 2017 Feb 20;22(2):325. doi: 10.3390/molecules22020325 (PMC6155910; doi:10.3390/molecules22020325)
Supplement: Supplementary file 1 [file molecules-22-00325-s001.pdf]

Supplementary Materials

Analysis of the Total Biflavonoids Extract from *Selaginella doederleinii* by HPLC-QTOF-MS and its *in vitro* and *in vivo* Anticancer Effects

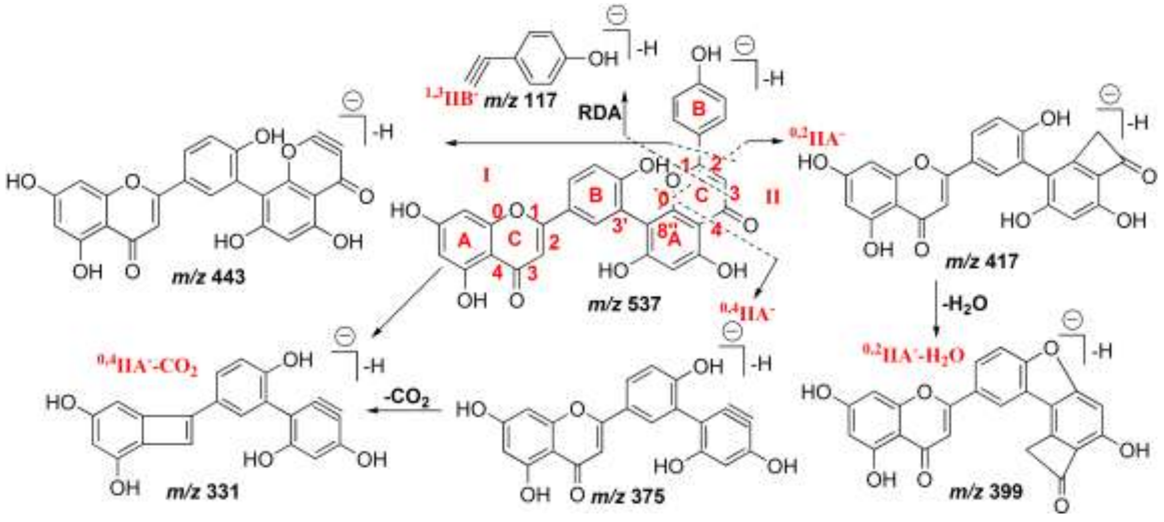

Figure S1. Proposed fragmentation pathway of amentoflavone in (–)ESI mode.

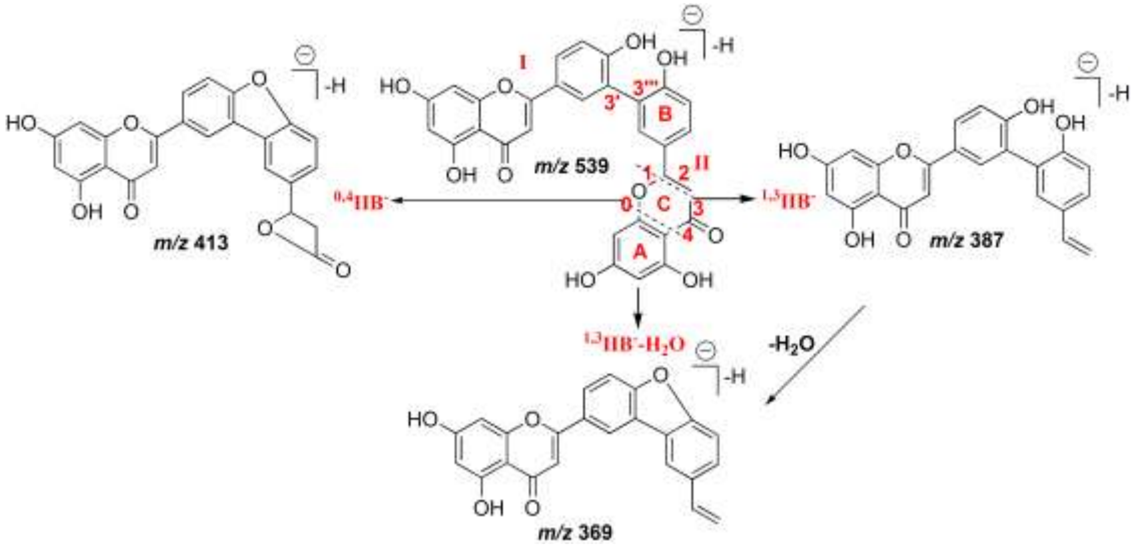

Figure S2. Proposed fragmentation pathway of 2′′,3′′-dihydrogen-3′,3′′′-biapigenin in (–)ESI mode.

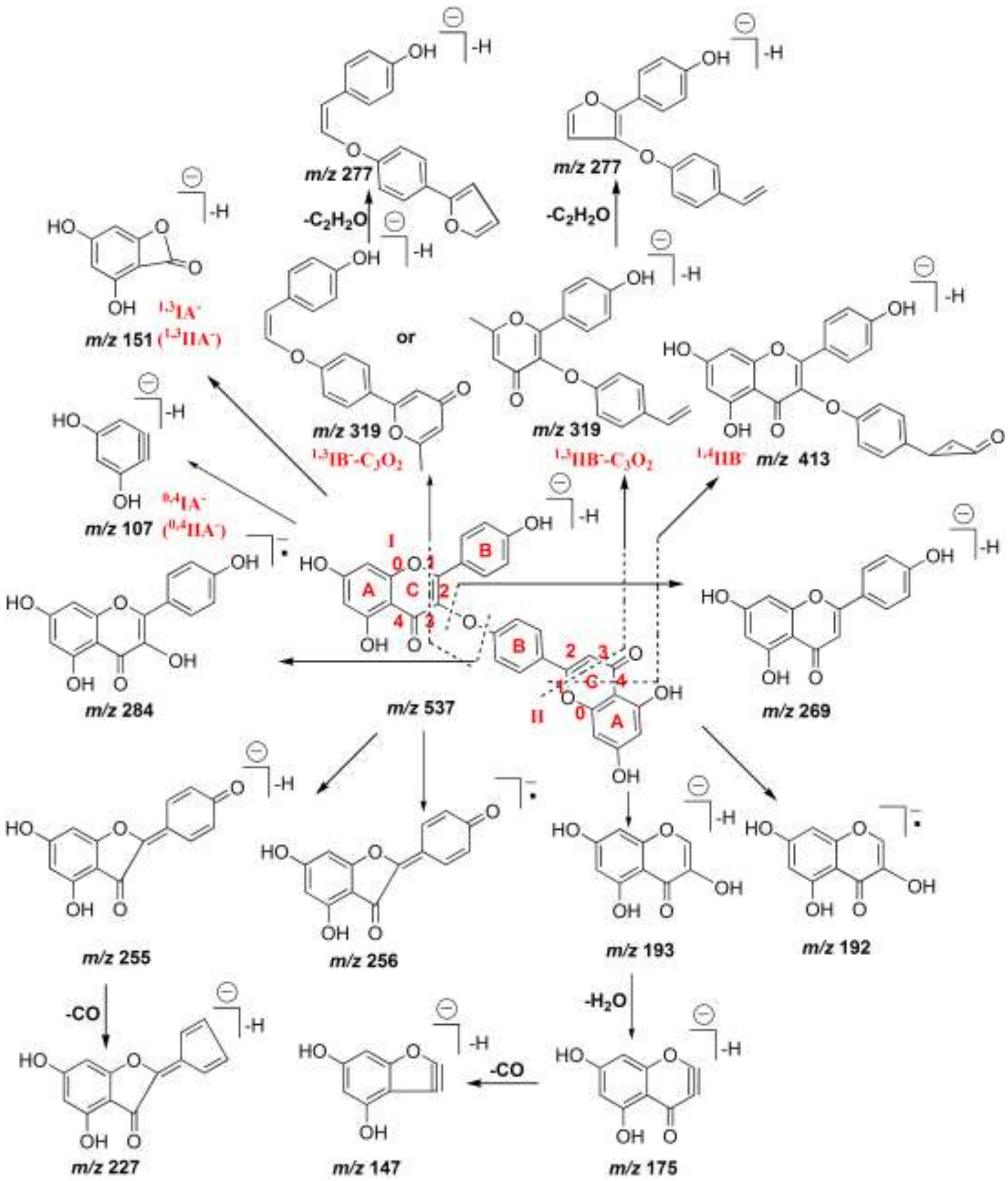

Figure S3. Proposed fragmentation pathway of delicaflavone in (–)ESI mode.

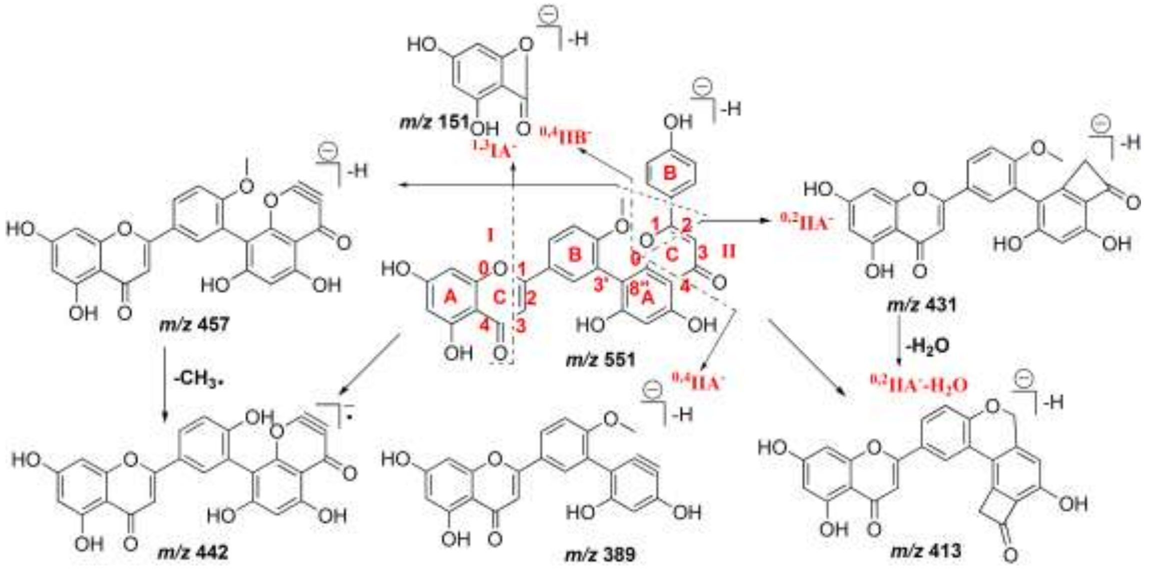

Figure S4. Proposed fragmentation pathway of bilobetin in (–)ESI mode.

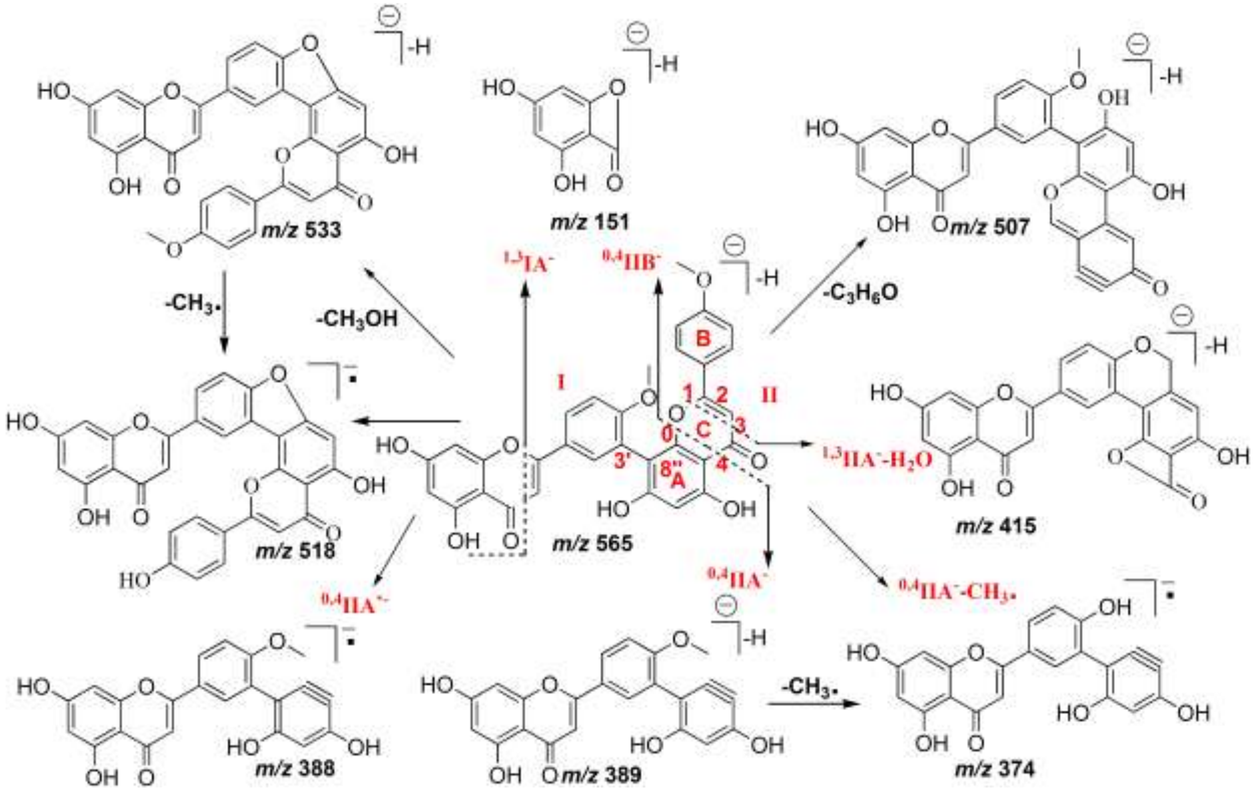

Figure S5. Proposed fragmentation pathway of isoginkgetin in (–)ESI mode.

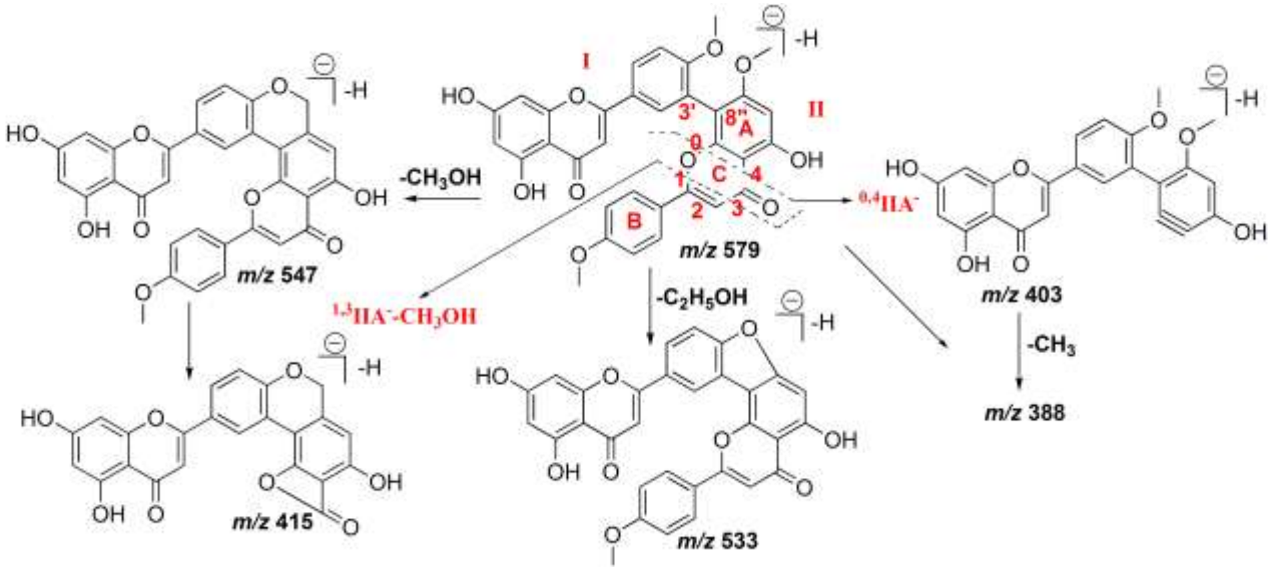

Figure S6. Proposed fragmentation pathway of kayaflavone in (–)ESI mode.

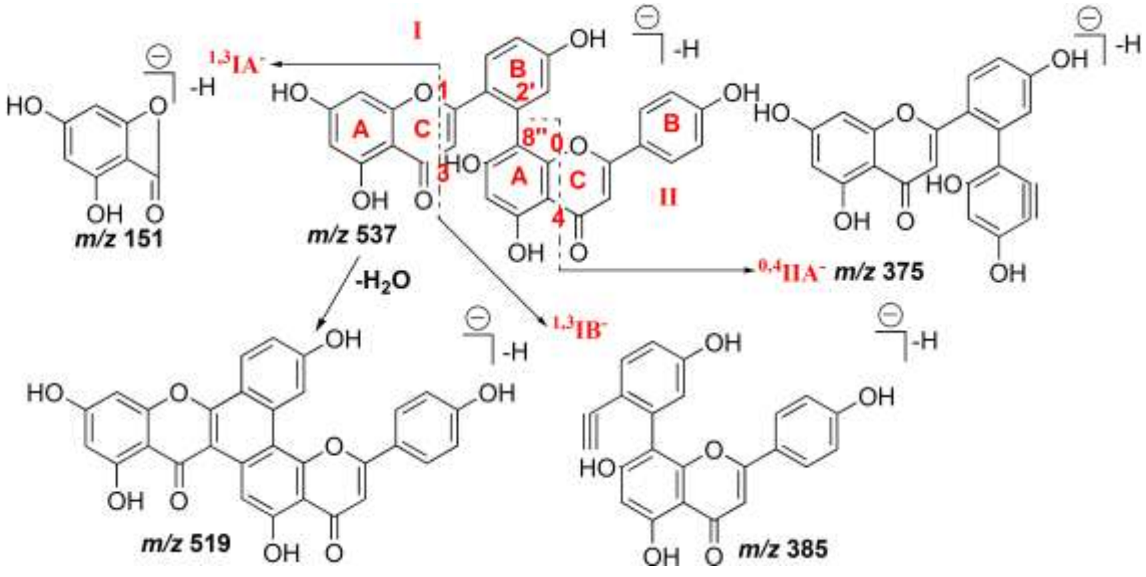

Figure S7. Proposed fragmentation pathway of 2',8''-biapigenin in (–)ESI mode.

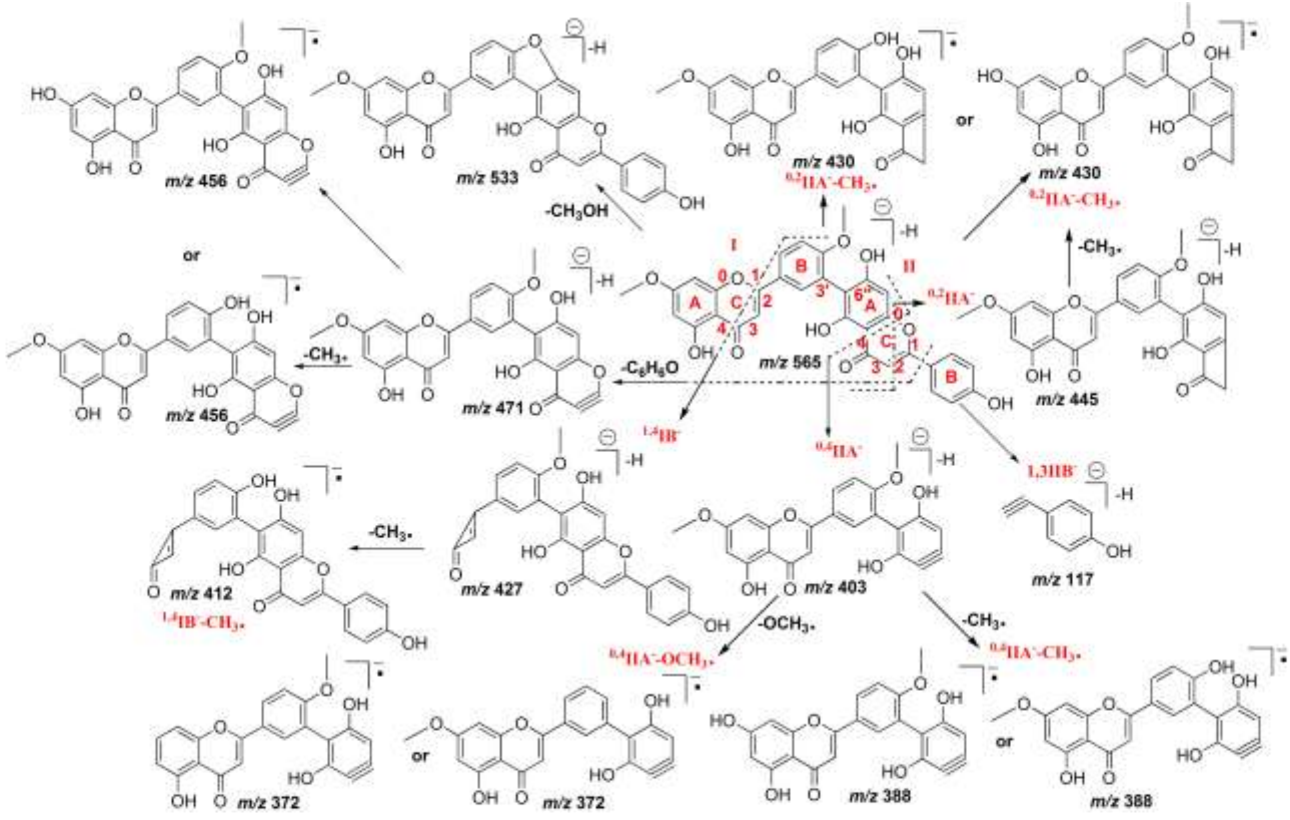

Figure S8. Proposed fragmentation pathway of robustaflavone 7,4'-dimethyl ether in (–)ESI mode.

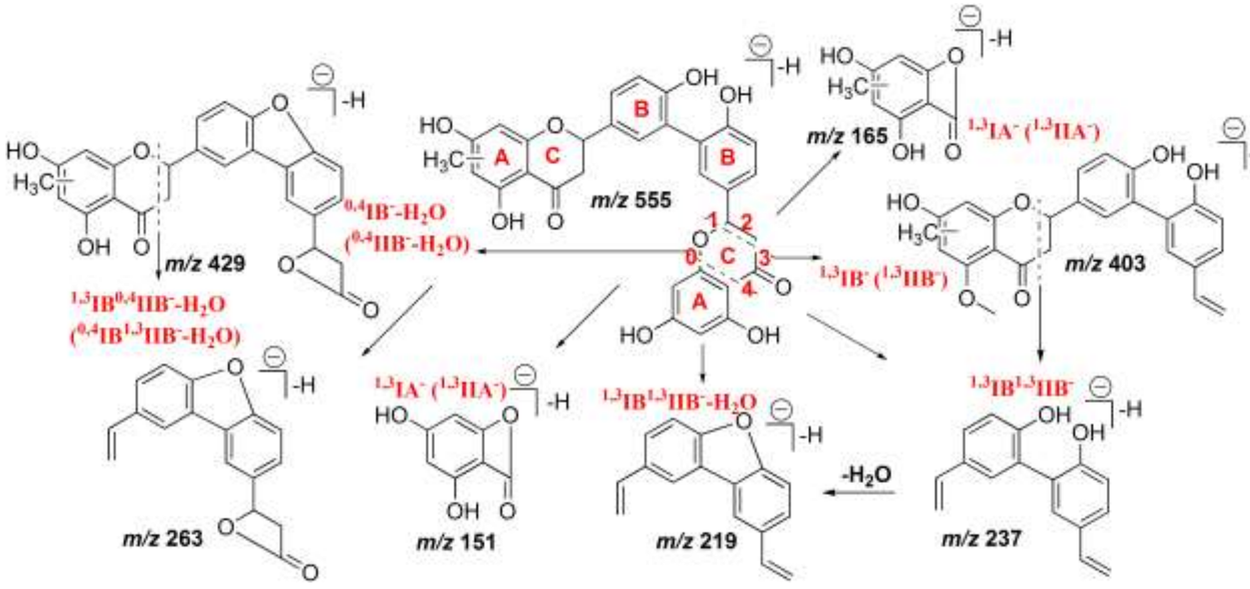

Figure S9. Proposed fragmentation pathway of 3',3'''-binaringen methyl ether in (–)ESI mode.

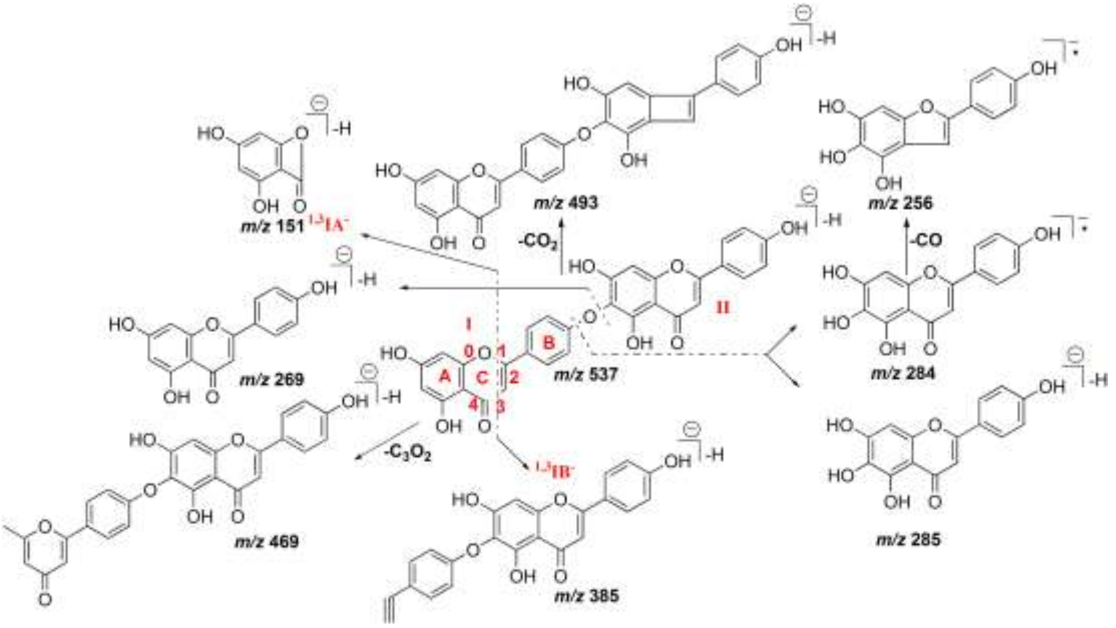

Figure S10. Proposed fragmentation pathway of hinokiflavone in (–)ESI mode.

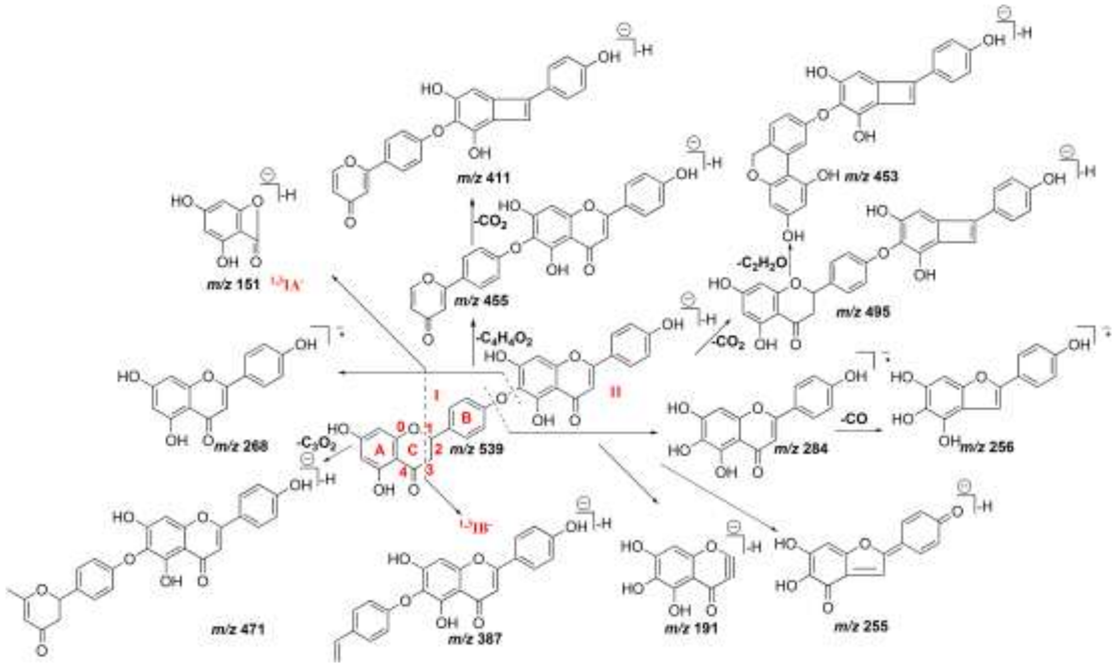

Figure S11. Proposed fragmentation pathway of 2,3-dihydrohinokiflavone in (–)ESI mode.

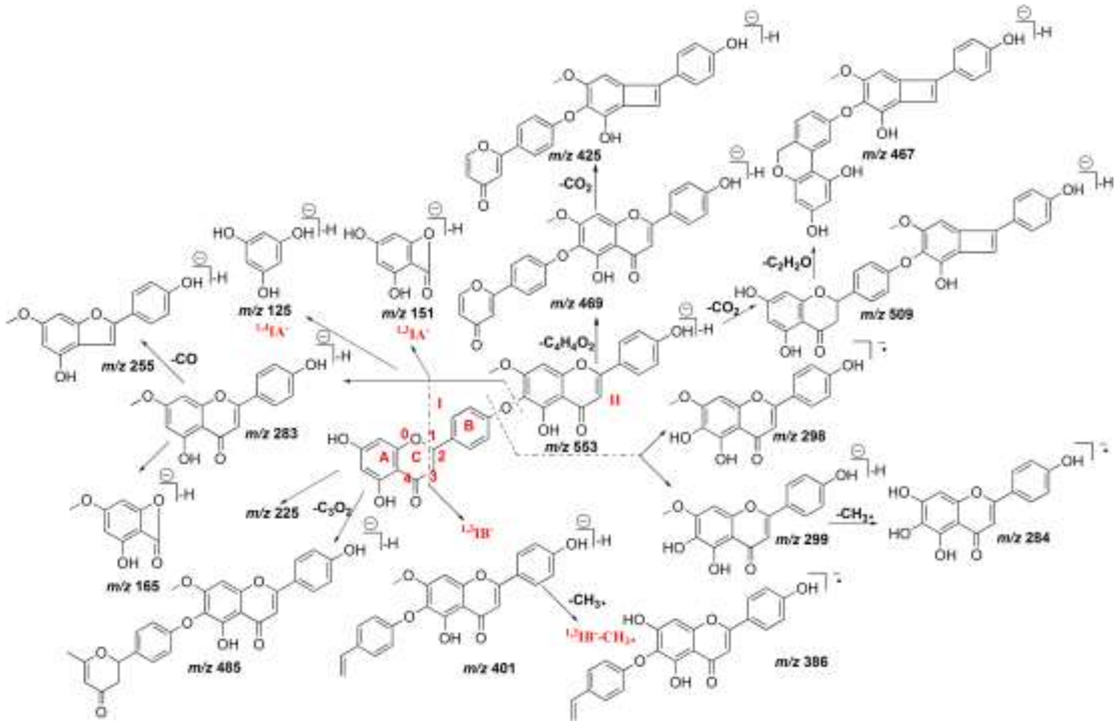

Figure S12. Proposed fragmentation pathway of 2,3-dihydroisocryptomerin in (–)ESI mode.

TableS 1. Q-TOF MS/MS data in (–)ESI mode of eight reference compounds.

**(–) ESI-MS^2 *m/z* (% base peak)**			**(–) ESI-MS^2 *m/z* (% base peak)**		
Observed mass	Calculated mass (Δppm)	Proposed formula	Observed mass	Calculated mass (Δppm)	Proposed formula
**Amentoflavone**			**Robustaflavone**		

MS²[537]:			MS²[537]:		
375.0507 (100)	375.051 (−0.80)	$C_{21}H_{11}O_7^-$	309.04 (100)	309.0405 (−1.62)	$C_{17}H_9O_6^-$
417.0613 (29)	417.0616 (−0.72)	$C_{23}H_{13}O_8^-$	537.0817 (96)	537.0827 (−1.86)	$C_{30}H_{17}O_{10}^-$
331.0608 (18)	331.0612 (−1.21)	$C_{20}H_{11}O_5^-$	375.0505 (70)	375.051 (1.33)	$C_{21}H_{11}O_7^-$
399.0507 (18)	399.051 (−0.75)	$C_{23}H_{11}O_7^-$	331.0607 (38)	331.0612 (−1.51)	$C_{20}H_{11}O_5^-$
537.0818 (8)	537.0827 (−1.68)	$C_{30}H_{17}O_{10}^-$	251.0345 (22)	251.035 (−1.99)	$C_{15}H_7O_4^-$
443.0403 (8)	443.0409 (−1.35)	$C_{24}H_{11}O_9^-$	387.0868 (21)	387.0874 (−1.55)	$C_{23}H_{15}O_6^-$
117.0343 (5)	117.0346 (−2.56)	$C_8H_5O^-$	225.0552 (20)	225.0557 (−2.22)	$C_{14}H_9O_3^-$
**3',3'''-Binaringenin**			117.0343 (20)	117.0346 (−2.56)	$C_8H_5O^-$
MS²[541]:			293.045 (19)	293.0455 (−1.71)	$C_{17}H_9O_5^-$
237.0919 (100)	237.0921 (−0.84)	$C_{16}H_{13}O_2^-$	413.0656 (19)	413.0667 (−2.66)	$C_{24}H_{13}O_7^-$
151.0036 (80)	151.0037 (−0.66)	$C_7H_3O_4^-$	417.0608 (18)	417.0616 (−1.92)	$C_{23}H_{13}O_8^-$
263.0712 (34)	263.0714 (−0.76)	$C_{17}H_{11}O_3^-$	519.0708 (17)	519.0722 (−2.70)	$C_{30}H_{15}O_9^-$
415.0815 (9)	415.0823 (−1.93)	$C_{24}H_{15}O_7^-$	**2'',3''-Dihydro-3',3'''-biapigenin**		
389.1022 (8)	389.1031 (−2.31)	$C_{23}H_{17}O_6^-$	MS²[539]:		
107.0137 (6)	107.0139 (−1.87)	$C_6H_3O_2^-$	387.0867 (100)	387.0874 (−1.81)	$C_{23}H_{15}O_6^-$
125.0242 (4)	125.0244 (−1.60)	$C_6H_5O_3^-$	413.0659 (9)	413.0667 (−1.94)	$C_{24}H_{13}O_7^-$
219.081 (4)	219.0815 (−2.28)	$C_{16}H_{11}O^-$	369.0762 (8)	369.0768 (−1.63)	$C_{23}H_{13}O_5^-$
371.0917 (4)	371.0925 (−2.16)	$C_{23}H_{15}O_5^-$	151.0036 (4)	151.0037 (−0.66)	$C_7H_3O_4^-$
**Delicaflavone**			**Chrysocauloflavone I**		
MS²[537]:			MS²[539]:		
193.0138 (100)	193.0142 (−2.07)	$C_9H_5O_5^-$	495.1069 (100)	495.1085 (−3.23)	$C_{29}H_{19}O_8^-$
537.0820 (97)	537.0827 (−1.30)	$C_{30}H_{17}O_{10}^-$	387.0864 (73)	387.0874 (−2.84)	$C_{23}H_{15}O_6^-$
192.0061 (87)	192.0064 (−1.56)	$C_9H_4O_5^{-\bullet}$	453.0964 (39)	453.0980 (−3.53)	$C_{27}H_{17}O_7^-$
151.0034 (70)	151.0037 (−1.99)	$C_7H_3O_4^-$	284.032 (28)	284.0326 (−2.11)	$C_{15}H_8O_6^{-\bullet}$
269.0452 (49)	269.0455 (−1.12)	$C_{15}H_9O_5^-$	285.0389 (28)	285.0405 (−5.61)	$C_{15}H_{19}O_6^-$
284.032 (43)	284.0326 (−2.11)	$C_{15}H_8O_6^{-\bullet}$	151.0032 (28)	151.0037 (−3.31)	$C_7H_3O_4^-$
319.0969 (34)	319.0976 (−2.19)	$C_{20}H_{15}O_4^-$	455.0778 (25)	455.0772 (1.32)	$C_{26}H_{15}O_8^-$
255.0298 (27)	255.0299 (−0.39)	$C_{14}H_7O_5^-$	125.0239 (19)	125.0244 (−4.00)	$C_6H_5O_3^-$

256.0365 (24)	256.0377 (–4.69)	$C_{14}H_8O_5^{-\bullet}$	539.0974 (17)	539.0984 (–1.85)	$C_{30}H_{19}O_{10}^{-}$
147.0083 (21)	147.0088 (–3.40)	$C_8H_3O_3^{-}$	371.091 (15)	371.0925 (–4.04)	$C_{23}H_{15}O_5^{-}$
175.0033 (20)	175.0037 (–2.29)	$C_9H_3O_4^{-}$	471.1068 (13)	471.1085 (–3.61)	$C_{27}H_{19}O_8^{-}$
107.0138 (17)	107.0139 (–0.93)	$C_6H_3O_2^{-}$	411.0875 (8)	411.0874 (0.24)	$C_{25}H_{15}O_6^{-}$
277.0864 (17)	277.087 (–2.17)	$C_{18}H_{13}O_3^{-}$	429.0968 (8)	429.098 (–2.80)	$C_{25}H_{17}O_7^{-}$
413.0664 (14)	413.0667 (–0.73)	$C_{24}H_{13}O_7^{-}$	256.0376 (7)	256.0377 (–0.39)	$C_{14}H_8O_5^{-\bullet}$
227.0344 (14)	227.035 (–2.64)	$C_{13}H_7O_4^{-}$			
**Heveaflavone**			**7,4',7'',4'''-Tetra-*O*-methyl-amentoflavone**		
MS^2[579]:			MS^2[593]:		
403.0818 (100)	403.0823 (–1.24)	$C_{23}H_{15}O_7^{-}$	563.1014	563.0984 (5.33)	$C_{32}H_{19}O_{10}^{-}$
388.0583 (68)	388.0589 (–1.55)	$C_{22}H_{12}O_7^{-\bullet}$	547.1061	547.1035 (4.75)	$C_{32}H_{19}O_9^{-}$

Table S2. Effect of the biflavonoids extract of *S. doederleinii* on tumor growth in male C57BL/6 mice with intragastric administration (mg/kg/d) (mean ± SD).

Groups	Animal number	Body weight (g) Before xenograft / After the treatment of 12 day	Tumor weight (g)	Inhibition rates (%)
Model	10	20.66±1.28/23.41±1.05	2.17±0.42	—
ADM (5mg/kg)	10	20.47±1.12/23.35±1.75	1.07±0.18*	50.92
The total biflavones extract (50mg/kg)	10	20.96 ±1.59/23.61±0.82	1.30±0.25*	40.11
The total biflavones extract (150mg/kg)	10	20.22±1.31/23.14±1.00	1.01±0.23*	53.50

Note: * $P < 0.01$, vs. Model group

Table S3. MVD in tumor issues at magnification 400×

Groups	Model	The total biflavones extract (50mg/kg)	The total biflavones extract (150mg/kg)
MVD	71.29±1.11	66.14±2.34 *	58.29±2.50 *

Note: * $P < 0.01$, vs. Model group

Table S4. Effects of the total biflavonoids extract of *S. doederleinii* on serum TNF-α and IFN-γ levels of LLC xenograft-tumor mice

Groups	Animal number	IFN-γ（ng/mL）	TNF-α（ng/mL）

Model	10	0.137±0.05	0.118±0.05
ADM (5mg/kg)	10	0.191±0.122	0.151±0.07 *
The total biflavones extract (50mg/kg)	10	0.152±0.09*	0.131±0.04*
The total biflavones extract (150mg/kg)	10	0.475±0.273$^{\triangle}$	0.369±0.10**

Note: * $P < 0.05$, vs. Model group; $^{\triangle}P < 0.01$, vs. Model group and low-dose group; ** $P < 0.01$, vs. Model group, ADM group and low-dose group.
